# Phylogenetic relationships and critical taxonomic notes on the genus *Astragalus* (Fabaceae) in Kazakhstan (Central Asia): Part I

**DOI:** 10.3897/phytokeys.274.180290

**Published:** 2026-05-08

**Authors:** Dariganga Munkhtulga, Shukherdorj Baasanmunkh, Jong Ho Park, Nudkhuu Nyamgerel, Serik Kubentayev, Daniyar Alibekov, Saule Mukhtubayeva, Zhansaya Idrissova, Alina Urazalina, Hyeok Jae Choi

**Affiliations:** 1 Department of Biology and Microbiology, Changwon National University, Changwon 51140, Republic of Korea Laboratory of NatureLaB, Astana International University Astana Kazakhstan; 2 Laboratory of NatureLaB, Astana International University, Astana 010000, Kazakhstan Department of Biology and Microbiology, Changwon National University Changwon Republic of Korea; 3 Astana Botanical Garden, Astana 010000, Kazakhstan Astana Botanical Garden Astana Kazakhstan; 4 Institute of Botany and Phytointroduction, Almaty 050000, Kazakhstan Institute of Botany and Phytointroduction Almaty Kazakhstan

**Keywords:** *
Astragalus
*, Central Asia, DNA barcoding, phylogeny, section, taxonomy

## Abstract

In the case of many previously described *Astragalus* species, more than 3,100 species worldwide, particularly those that are endemic and rare, there is a lack of critical information, such as wild photo illustrations, taxonomic notes, and genetic resources. We investigated the taxonomic status and phylogenetic relationships of 26 *Astragalus* species including 3 endemics to Kazakhstan, using samples collected in 2025 to address this gap. We sequenced the internal transcribed spacer (ITS) of all studied *Astragalus* species. In the phylogenetic tree, all *Astragalus* species were monophyletic and had the same topology as reported in previous studies. Our phylogenetic tree was divided into two primary clades. Clade I included species of the subgenera *Calycophysa* and *Caprinus*, whereas Clade II included species of the subgenera *Cercidothrix* and *Trimeniaeus*. In Clade I, each section was well-separated from the others, except for the sections *Chaetodon*, *Erioceras*, and *Dissitiflori* in the subg. *Cercidothrix*. The taxonomic positions of some *Astragalus* species, based on morphological and molecular evidence, are also discussed. The detailed illustrations and species documentation presented in this study will serve as significant references for future taxonomic and identification studies of this genus.

## Introduction

The genus *Astragalus* L. (Fabaceae) is one of the largest and most diverse genera of angiosperm, comprising more than 3,100 species from 250 sections worldwide (Podlech and Zarre 2013; POWO 2025). Several fundamental taxonomic treatments of *Astragalus* have been published (Ulziykhutag 2004; Xu and Podlech 2010; Podlech and Zarre 2013), however, many species remain poorly understood because of their morphological similarities and overlapping characteristics. Recent advances in molecular phylogenetics have provided new insights into the relationships within this genus, prompting the need for comprehensive taxonomic revisions (Azani et al. 2017, 2019; Folk et al. 2024; Buono et al. 2025). Morphologically, *Astragalus* species are highly similar to other members of the Astragalean clade, such as the genus *Oxytropis* DC. The high diversity richness of this genus is frequently distributed in the Old World’s countries (Sanderson and Wojciechowski 1996; Maassoumi and Ashouri 2022) including Iran (ca. 850 species), Turkiye (ca. 479 species) (Aytaç 2000; Uzun et al. 2021), China (ca. 400 species) (Xu and Podlech 2010), Uzbekistan (ca. 273 species) (Tojibaev et al. 2015; Sennikov et al. 2016), Pakistan (ca. 150 species) (Khan et al. 2023), Kyrgyzstan (ca. 189 species) (Sytin and Lazkov 2018; Mamatkulov et al. 2025), and Mongolia (ca. 130 species) (Baasanmunkh et al. 2024a; Munkhtulga et al. 2025).

Approximately 320 *Astragalus* species, belonging to 60 sections and 8 subgenera, have been recorded in Kazakhstan (Abdulina 1998; Perezhogin et al. 2023). Among these, 45 endemic species have been recognized, according to the latest checklist of endemic vascular plants (Kubentayev et al. 2024). Over the past two decades, five new species and one subspecies of *Astragalus* have been described in Kazakhstan: *A. aktiubensis* Sytin (Sytin 2000), *A. arcanus* Knjaz., Krivenko & E.G.Philippov (Knyazev 2019b), *A. austroaltaicus* Popov & Knjaz. (Knyazev 2016), *A. lagobromus* Knjaz. & Kulikov (Knyazev and Kulikov 2011), *A. saphronovae* Kulikov (Kulikov 2014), *A. kasachstanicus* subsp. *coloratus* Knjaz. (Knyazev 2019a). In addition, one species previously unknown to Kazakhstan’s flora has been recorded (Kupriyanov et al. 2024), and several taxa have been identified in certain regions of the country (Perezhogin et al. 2023). Several studies have examined the taxonomic revision and distribution of *Astragalus* species in Kazakhstan based solely on morphological evidence (Knyazev 2019a; Kotukhov 2020; Perezhogin et al. 2023; Kupriyanov et al. 2024; Kubentayev et al. 2024). These findings indicate that the diversity of *Astragalus* in Kazakhstan has not been adequately studied and highlight the need for further taxonomic, floristic, and geobotanical research to reveal the true species richness of the genus and refining its distribution within the natural zones of the Republic.

Recently, a large number of photo observations of *Astragalus* species have been uploaded by citizen scientists to the “Flora of Kazakhstan” project (https://www.inaturalist.org/projects/flora-of-kazakhstan-aza-stan-florasy) on the iNaturalist platform. This is because iNaturalist is among the most widely used citizen science platforms globally for encouraging public participation in citizen science and promoting biodiversity conservation (Richardson and Potgieter 2024; Mesaglio et al. 2025; Mason et al. 2025; Baasanmunkh et al. 2026). However, many of these *Astragalus* observations have not been fully identified. They are marked as “Needs ID” due to taxonomic difficulties and the fact that they are based on single wild photographs. For example, complete taxonomic identification of *Astragalus* requires detailed morphological characteristics such as flower size (e.g., standard, wings, and keel), shape, and hair of pods and leaves (Podlech and Zarre 2013; Baasanmunkh et al. 2024a; Munkhtulga et al. 2025).

DNA barcoding of plants typically uses regions of the chloroplast genome, such as *rbcL* and *matK*, and an internal transcribed spacer (ITS), to identify and differentiate species. This method is a reliable tool for plant taxonomy, biodiversity assessment, and conservation efforts (Erst et al. 2022). Numerous studies have been conducted on the *Astragalus* genus using ITS, *matK* and *rbcL* markers (Wojciechowski et al. 1999; Dong et al. 2003; Kang et al. 2003; Kazempour Osaloo et al. 2003, 2005; Uzun et al. 2009; Gao et al. 2009; Kazemi et al. 2009; Zhang and Jiang 2020; Bagheri et al. 2022, 2023; Erst et al. 2022; Karaman Erkul et al. 2022; Baasanmunkh et al. 2024a; Munkhtulga et al. 2025) and chloroplast genome sequences based on the genome skimming approach (Su et al. 2021). Due to its size, morphological diversity, and frequent taxonomic complexity, *Astragalus* is a challenging group for traditional classification, making DNA barcoding a valuable tool. Most barcoding studies on *Astragalus* species have been conducted in Asia including China, Iran, and Mongolia (Kazempour Osaloo et al. 2003, 2005; Zhang and Jiang 2020; Bagheri et al. 2023; Baasanmunkh et al. 2024a; Munkhtulga et al. 2025). According to their research, ITS markers have been successfully identified in most *Astragalus* species, outperforming the chloroplast region (*matK*) in terms of recognition (Bagheri et al. 2016; Zhang and Jiang 2020; Baasanmunkh et al. 2024a; Munkhtulga et al. 2025; Avazzadeh et al. 2025). Despite some research, the morphological diagnosis, DNA barcoding, and phylogenetic position of Kazakhstan *Astragalus* remain poorly understood.

In this study, we conducted a critical examination of 26 *Astragalus* species, including 3 endemics to Kazakhstan, integrating morphological and molecular data (nrITS) to clarify the species. Additionally, morphological photo illustrations and taxonomic diagnoses were based only on their collections during the field expeditions. These results contribute to a more robust classification and highlight the importance of integrative approaches for the *Astragalus* genus across Central Asia.

## Materials and methods

### Taxon sampling

**Figure 1.**
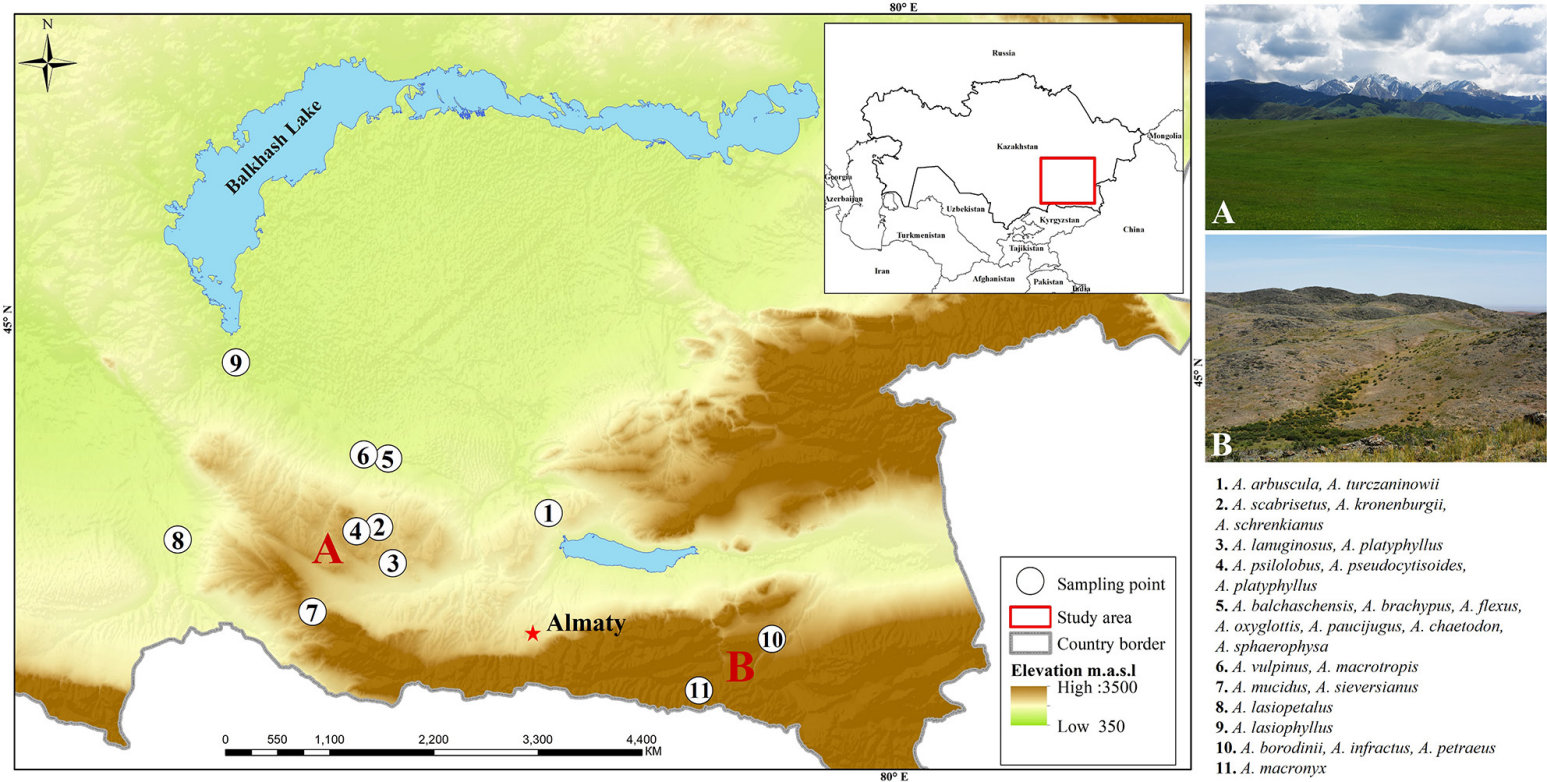
Map of field collection sites in Kazakhstan. Numbers are indicated representing collected species. General habitats of Chu-Ily mountains (**A**), Kolsay (**B**).

Field expeditions were conducted in two main areas, including the Chu-Ily and Kolsay mountains between 04 and 14 of May, 2025 (Fig. 1). We collected more than 30 *Astragalus* species, including detailed wild photographs, herbarium specimens, and fresh leaves, during field expeditions. The accepted names of each species were based on the Plants of the World Online (POWO 2025; http://www.plantsoftheworldonline.org/) and International Plant Name Index (IPNI 2025; www.ipni.org). The section and subgenus levels of each species were as described by Podlech and Zarre (2013). For species identification, we used various sources including literature (Abdulina 1998; Sytin 2000; Ulziykhutag 2004; Vydrina 2006; Xu and Podlech 2010; Podlech and Zarre 2013; Perezhogin et al. 2023), herbarium specimens from AA, BM, LE, MW (Thiers 2023), and occurrence records deposited in Plantarium (Plantarium 2025; http://www.plantarium.ru), “Global Biodiversity Information Facility” (GBIF 2025; https://www.gbif.org/), and iNaturalist (iNaturalist contributors 2025) for species identification. The ArcGIS 10.3 program was used for data mapping (Esri 2012). All collected specimens were deposited in the herbaria of the Astana Botanical Garden (NUR) and Changwon National University (CWNU).

### DNA extraction, amplification, and sequencing

Total genomic DNA was extracted from the silica gel–dried leaves following the CTAB protocol of Doyle and Doyle (1987). We isolated 54 DNA samples from 26 *Astragalus* species. At least two DNA samples were isolated from each species. Amplification and sequencing have focused on the nuclear ITS region (White et al. 1990). The PCR procedures followed those of Baasanmunkh et al. (2024a) and Munkhtulga et al. (2025), and the resulting amplicons were sequenced bidirectionally using BIONICS (Seoul, South Korea). Raw sequence data were examined and manually curated in Geneious Prime v.2025.2.2 (www.geneious.com). Multiple sequence alignments were performed using with MAFFT v.7.490 (Katoh and Standley 2013), and a consensus dataset was assembled from the aligned sequences. All newly generated sequences were deposited in GenBank (NCBI, www.ncbi.nlm.nih.gov).

### Phylogenetic analysis

A total of 60 accessions were included in the constructed ITS dataset, representing 28 *Astragalus* species and 2 *Phyllolobium* species including *P. chinense* Fisch. and *P. donianum* (DC.) M.L.Zhang & Podlech were selected as outgroups. Among the 58 accessions of *Astragalus*, 18 accessions belonging to 9 species were retrieved from GenBank (Table S1). Detailed information, including taxon names, accession numbers, collection sites, and references, is provided in Table S1. Phylogenetic analyses were performed using maximum parsimony (MP) in RAxML v.8.2.11 (Stamatakis 2014) as implemented in Geneious, employing the best-scoring maximum likelihood (ML) tree search and 1,000 bootstrap replicates. The reconstructed phylogenetic trees were visualized using FigTree v.1.4.2 (Rambaut 2012).

## Results

In this study, we investigated the taxonomic status and phylogenetic relationships of 26 *Astragalus* species from the Chu-Ily mountain and Kolsay lake ranges in Kazakhstan based on field expeditions. The higher taxonomic classification of 26 species belonged to 18 sections and 4 subgenera. Among these, we identified three endemic species: *A. balchaschensis* Sumnev, *A. pseudocytisoides* Popov, and *A. psilolobus* Puchkova. We provided taxonomic notes, photo illustration, global distribution, and habitat information for each species. In particular, the taxonomic notes of each species were compared with those of the most closely similar species.

### Phylogenetic relationship of *Astragalus* species in Kazakhstan

**Figure 2.**
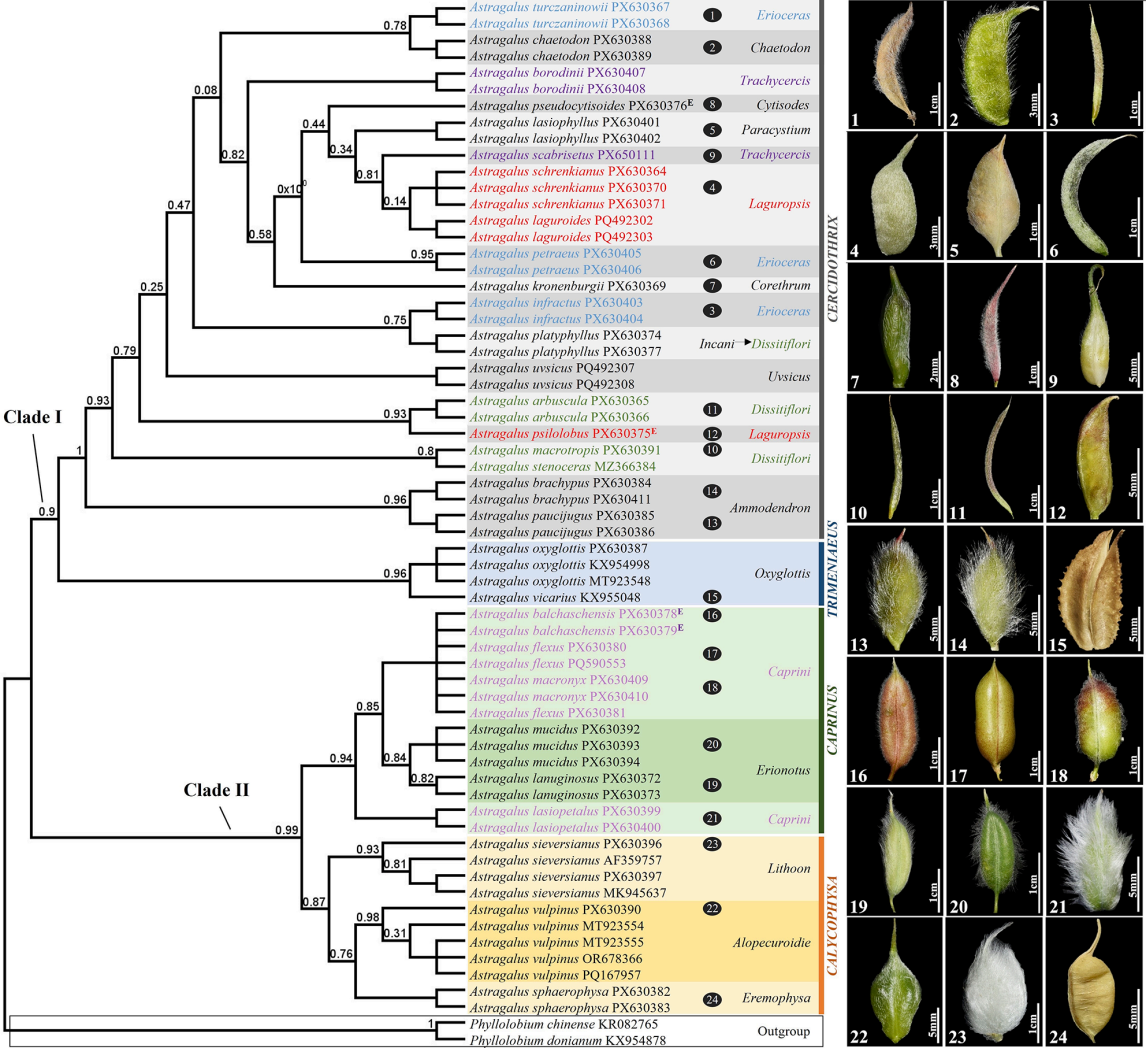
Phylogenetic tree of *Astragalus* species based on ITS sequences, with corresponding pod morphology. Species of subg. *Cercidothrix* are indicated by grey borders, followed by subg. *Trimeniaeus* by blue borders, subg. *Astragalus* by green borders, and subg. *Calycophysa* by orange borders. Species assigned to the same section by Podlech and Zarre (2013) are printed in the same colours on the tree.

We obtained ITS sequences from all accessions of the 26 *Astragalus* species with 100% success in both PCR and sequencing. The ITS matrix contained 744 bp and 8 indels; the distribution of 156 bp parsimony-informative sites and 160 bp variable sites was dense across the matrix. We compared our newly sequenced species, *A. vulpinus*, *A. sieversianus*, *A. oxyglottis*, and *A. flexus* to previous sequence data in NCBI, which were similar to each other (Fig. 2). The ITS sequences of the remaining 24 *Astragalus* species were sequenced from Kazakhstan for the first time. Our phylogenetic tree was divided into two primary clades. Clade I included species of the subgenera *Calycophysa* and *Caprinus*. Clade II included species of the subgenera *Cercidothrix* and *Trimeniaeus* (Fig. 2). In Clade I, each section was well-separated from the others, except for the sect. *Chaetodon*, *Erioceras*, and *Dissitiflori* in the subg. *Cercidothrix* (Fig. 2). In Clade II, sect. *Caprini* was not grouped into a single section in the subg. *Caprinus*. For the phylogenetic tree, we have added an illustration of the pods based on the species collected in the country (Fig. 2).

## Discussion

### Phylogenetic relationship of *Astragalus* species in Kazakhstan

The ITS region has been frequently used in phylogenetic research because of its high variation and optimal effectiveness in distinguishing closely related species (Yun et al. 2003; Gao et al. 2009; Zhang and Jiang 2020; Bagheri et al. 2022, 2023; Erst et al. 2022; Karaman Erkul et al. 2022; Baasanmunkh et al. 2024a; Munkhtulga et al. 2025). Furthermore, the ITS region showed a higher PCR amplification success rate and discriminatory ability than the chloroplast regions (*mat*K, *rbc*L, and *trn*H–*psb*A) (Zhang and Jiang 2020). All our studied species of *Astragalus* in the phylogenetic tree were monophyletic, similar to the results of previous studies (Yun et al. 2003; Gao et al. 2009; Zhang and Jiang 2020; Bagheri et al. 2022, 2023; Erst et al. 2022; Karaman Erkul et al. 2022; Baasanmunkh et al. 2024a; Munkhtulga et al. 2025).

Morphology-based classification showed considerable incongruence with the phylogenetic results in the subg. *Cercidothrix*. For example, *A. turczaninowii* was morphologically assigned to the sect. *Erioceras*, although phylogenetically, was more closely related to *A. chaetodon* of sect. *Chaetodon* (Fig. 2), and is closely related to *A. infractus* and *A. petraeus* of sect. *Erioceras* (Fig. 2). Similarly, *A. borodinii* and *A. scabrisetus*, though traditionally placed in the same section, form distinct and independent lineages. Similarly, *A. schrenkianus* and *A. psilolobus*, which belong to the same section morphologically, appeared at different positions in the molecular phylogeny. Interestingly, several earlier classification systems that predated that of Podlech and Zarre (2013) were more consistent with our molecular results. For example, *A. borodinii* was previously placed in sect. *Borodiniana* B.Fedtsch. (Ulziykhutag 2004), *A. scabrisetus* in sect. *Scabriseta* R.Kam. (Ulziykhutag 2004), *A. turczaninowii* in sect. *Tamias* Bunge (Puchkova 1967), and *A. lasiopetalus* in sect. *Erionotus* (Goncharov et al. 1946; Ulziykhutag 2004), arrangements that appear more congruent with the molecular evidence.

Several taxa, including *A. infractus*, *A. petraeus*, *A. platyphyllus*, and *A. psilolobus*, yielded noteworthy results. *Astragalus psilolobus* was originally described by Puchkova (1967) as a member of the sect. *Chaetodon*, who emphasized its distinctive morphological traits and suggested that its phylogenetic position should be verified using molecular data. Later, Podlech and Zarre (2013) transferred this species to the sect. *Laguropsis* based solely on descriptions. However, our molecular analysis revealed that *A. psilolobus* is genetically distant from both *Chaetodon* and *Laguropsis*, showing a closer affinity with *A. arbuscula* in the sect. *Dissitiflori*. This suggests that *A. psilolobus* represents an independent and potentially new section.

*Astragalus infractus* was most closely related to *A. platyphyllus*, whereas *A. petraeus* formed a distinct branch showing affinities with members of sect. *Cytisodes*, *Paracystium*, *Trachycercis*, and *Laguropsis*. *Astragalus platyphyllus* was originally assigned to the sect. *Incani*, but its taxonomic placement remains uncertain. In a phylogenetic study by Amini et al. (2019), based on combined ITS and *rpl32–trnL*(UAG) data, this species was found not to share a common origin with other members of sect. *Incani* but to cluster with representatives of sect. *Dissitiflori*, leading the authors to transfer it accordingly.

Within the *Astragalus* clade, the interspecific differentiation appeared relatively weak. Similar results have been observed in other studies; although morphological differences among species were evident, genetic divergence remained low. For example, Riahi et al. (2011) reported that ITS, ETS, and cpDNA (*matK* and *trnL–F*) markers exhibited minimal variation among species of sect. *Caprini*. They attributed this pattern to rapid diversification during the Late Miocene–Pliocene and incomplete lineage sorting (ILS). The authors also proposed that continuous hybridization and gene flow among species inhabiting arid montane ecosystems have blurred internal genetic boundaries. Similarly, Maylandt et al. (2025) found that European representatives of of sect. *Caprini* displayed very low differentiation at the nrDNA and cpDNA levels despite marked morphological distinctions. They explained this pattern as a combined outcome of rapid Pleistocene diversification, geographic isolation, climatic oscillations, hybridization, and chloroplast capture. Therefore, the limited genetic differentiation observed within the *Astragalus* clade likely reflects a combination of recent rapid speciation, introgressive hybridization, and ongoing gene flow. The pronounced morphological divergence among species may represent instances of parallel evolution driven by ecological selection pressures.

### Taxonomic treatment of 26 *Astragalus* species in Kazakhstan

#### *Astragalus* subgenus *Caprinus* Bunge, Astrag. turk 218 (1880).

**Sect. *Caprini* DC., Prodr. 2: 301 (1825)**.

##### Astragalus balchaschensis

Taxon classification

Plantae

FabalesFabaceae

Sumnev., Sist. Zametki Mater. Gerb. Krylova Tomsk. Gosud. Univ. Kuybysheva 1933(1–2): 4 (1933).

A3EB28A9-8CF9-5E22-86CA-98E056DB9026

Fig. 3

###### Notes.

This species is locally endemic to Balkhash Lake in southeastern Kazakhstan (Kubentayev et al. 2024). We found this species (voucher number: MT5-1) in sandy and sandy-pebbly steppes of the Chu-Ily Mountain range, occurring at elevations of 400–420 m. The morphological characteristics of this species are very similar to that of *A. buchtormensis* Pall., but it can be distinguished by its leaf (5–)8–15(–25) cm long; 13–18(–22) pairs; leaflets ovate to obovate, 5–8 × 3–5 mm (vs. (8–)10–20(–30) cm long; 8–30 pairs, leaflets narrowly oblong to narrowly ovate, 5–12 × 2–4 mm), pods 14–25 × 6–8 mm, acuminate with a beak 2–3 mm, densely hairy (vs. 15–20 × 6–8 mm, with a beak 3–5 mm, loosely hairy or sometimes glabrous).

**Figure 3.**
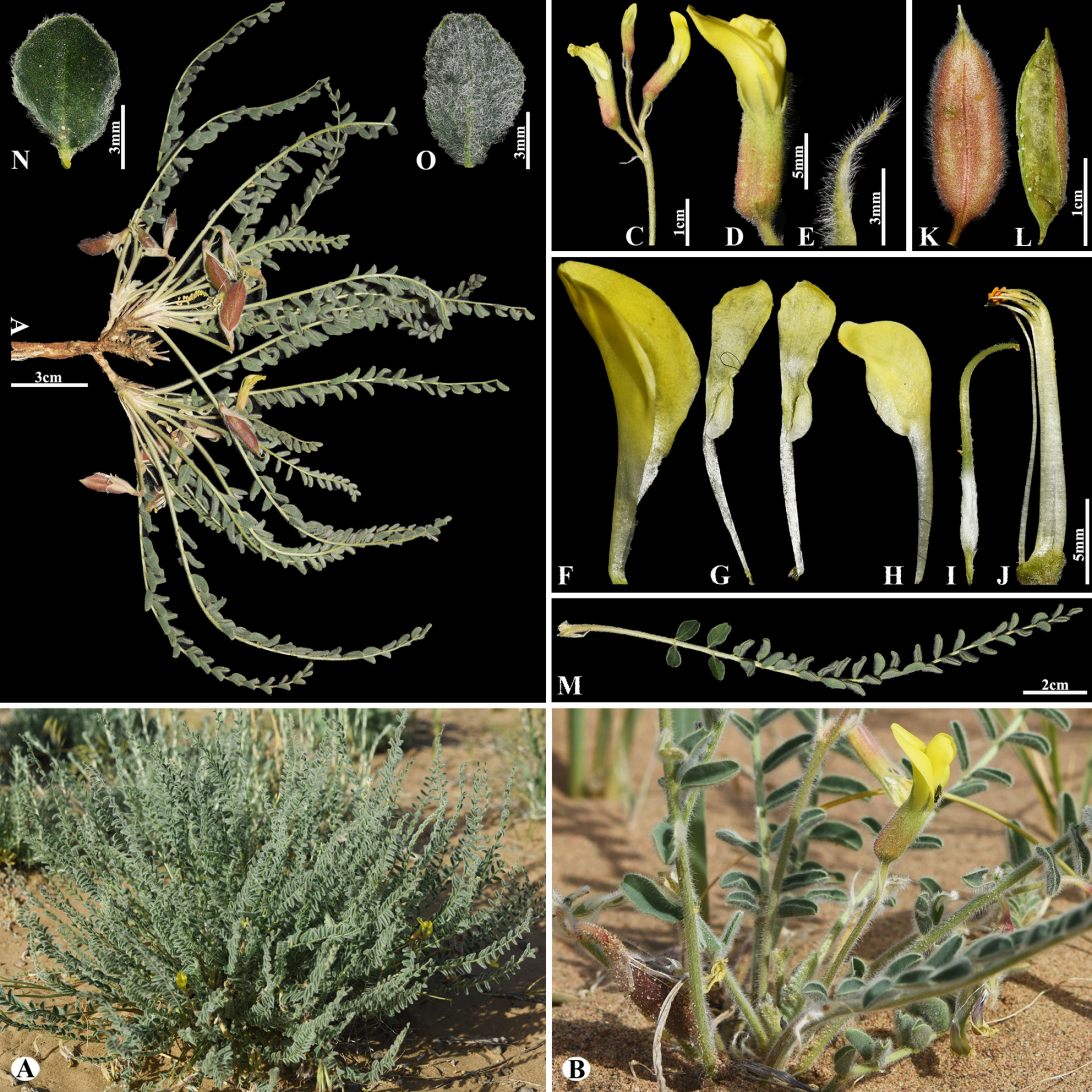
*Astragalus balchaschensis* in Kazakhstan (Voucher number: MT5-1). **A**. General habit; **B**. Peduncle; **C**. Raceme; **D**. Flower; **E**. Bract; **F**. Standard; **G**. Wings; **H**. Keel; **I**. Pistil; **J**. Stamens; **K**. Pod; **L**. Pod valve; **M**. Leaf; **N**. Leaflet, adaxial view; **O**. Leaflet, abaxial view. (Photo credits: D.Munkhtulga).

##### Astragalus flexus

Taxon classification

Plantae

FabalesFabaceae

Fisch., Bull. Cl. Phys.-Math. Acad. Imp. Sci. Saint-Pétersbourg 3: 307 (1845).

BEA667ED-445B-5255-A3FC-2FC8B148D7A0

Fig. 4

≡ Tragacantha flexa (Fisch.) Kuntze, Revis. Gen. Pl. 2: 945 (1891).
= Astragalus aquae-rubrae B.Fedtsch., Beih. Bot. Centralbl. 22(2): 352 (1908).
= Astragalus pentapetaloides Bunge, Beitr. Fl. Russl.: 98 (1852).
= Astragalus pentapetaloides var. *blepharophyllus* Bunge, Beitr. Fl. Russl.: 98 (1852).
= Astragalus remotijugus var. *pumilus* Parsa, Fl. Iran 9: 64 (1966).
= Astragalus stenanthus Freyn, Bull. Herb. Boissier, sér. 2, 4: 761 (1904), nom. illeg.

###### Notes.

This species is distributed throughout Russia (Europe), Kazakhstan, Uzbekistan, Turkmenistan, Tajikistan, Iran and China (POWO 2025). We found this species (voucher number: MT5-7) in hummocky and fixed sandy areas of the Chu-Ily Mountain range, occurring at elevations of 500–550 m. Morphologically, *A. flexus* is similar to *A. longipetalus* Chater, however, it differs in that its leaflet is ovate to obovate, 1.5–2× longer than wide. According to previous studies, phylogenetic analyses (ITS) revealed weak resolution among species of sect. *Caprini*, because of rapid and/or very recent diversification (Scherson et al. 2008; Zhang et al. 2009; Maylandt et al. 2025). Similarly, the studied species were not well separated from one another within sect. *Caprini* (Fig. 2).

**Figure 4.**
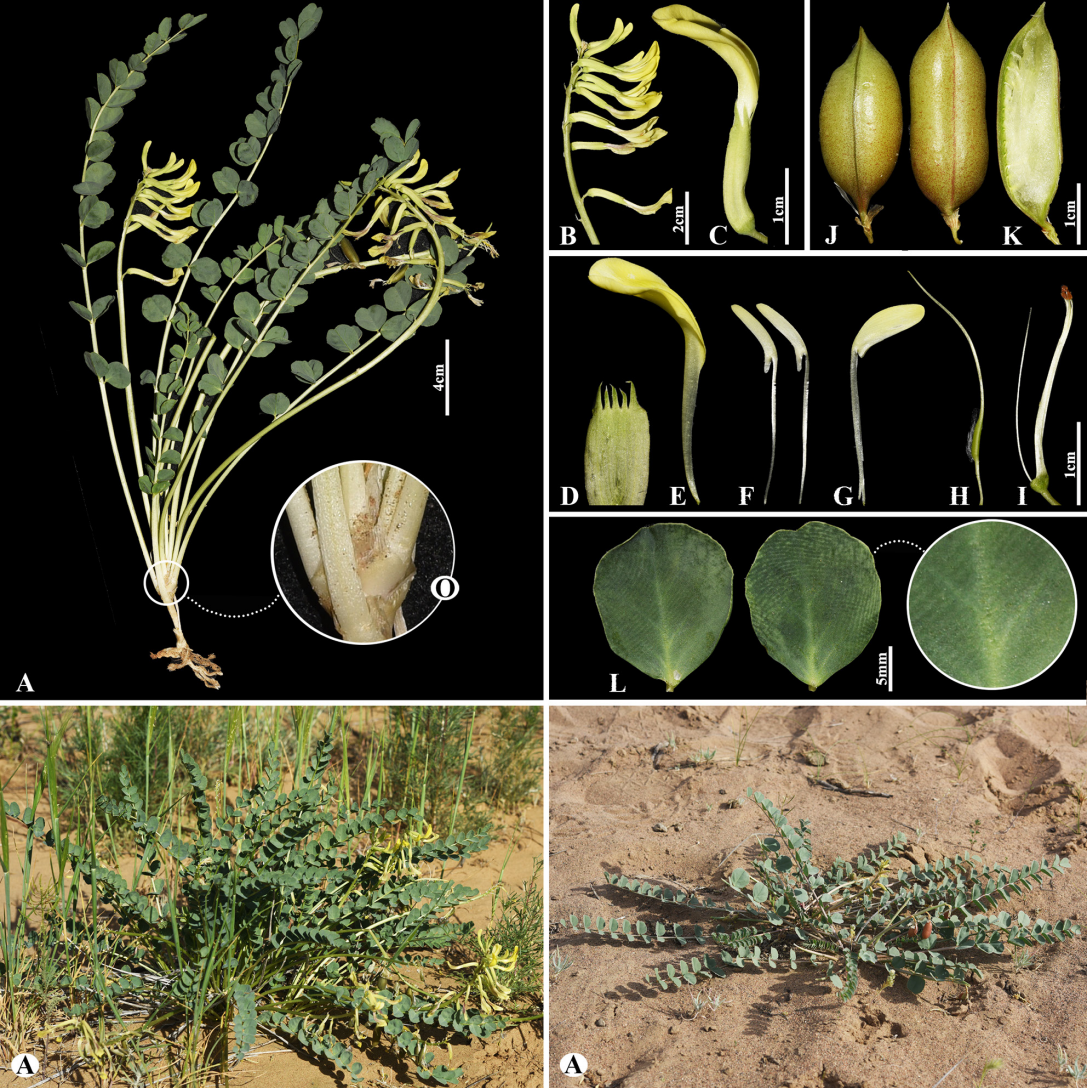
*Astragalus flexus* in Kazakhstan (Voucher number: MT5-7). **A**. General habit; **B**. Raceme; **C**. Flower; **D**. Calyx; **E**. Standard; **F**. Wings; **G**. Keel; **H**. Pistil; **I**. Stamens; **J**. Pod; **K**. Pod valve; **L**. Leaflet. (Photo credits: D.Munkhtulga).

##### Astragalus lasiopetalus

Taxon classification

Plantae

FabalesFabaceae

Bunge, Index Seminum (TU, Dorpatensis) 1839: 7 (1841).

7E9C4924-F9B9-54FA-8D52-8C0204465304

Fig. 5

≡ Myobroma lasiopetala (Bunge) Steven, Bull. Soc. Imp. Naturalistes Moscou 29(2): 150 (1856).
≡ Tragacantha lasiopetala (Bunge) Kuntze, Revis. Gen. Pl. 2: 946 (1891).
= Astragalus lasianthus C.A.Mey., A.G.H.Bongard & C.A.Meyer, Fl. Altaic., Suppl. 2: 92 (1841).
= Astragalus ulacholensis B.Fedtsch., Trudy Imp. S.-Peterburgsk. Bot. Sada 24: 208 (1905).

###### Notes.

This species is distributed throughout Kazakhstan, Kyrgyzstan, Mongolia, Tajikistan, China (Xinjiang), and Uzbekistan (Podlech and Zarre 2013). We found this species (voucher number: MT9) in meadows and solonchak banks of rivers of the Chu River, Shu district, occurring at elevations of 410–460 m. *Astragalus lasiopetalus* belongs to the sect. *Erionotus* (Ulziykhutag 2003), however, this section was transferred to the sect. *Caprini*, according to Podlech and Zarre (2013). In this study, *A. lasiopetalus* was clustered with the sect. *Erionotus* based on phylogenetic results (Fig. 2). *Astragalus lasiopetalus* is morphologically similar to *A. lanuginosus*, but can be distinguished by its leaflets (7–)9–12(–15) pairs, ovate to elliptic, 10–25(–32) × 7–16(–20) mm, upper side glabrous or nearly so, underside with ascending hairs (vs. (16–)20–26 pairs, ovate to elliptic or obovate, (4–)5–12 × (3–)4–8 mm, upper side cotton-wool like, often glabrescent, underside subappressed hairy), racemes 2–10(–12)-flowered (vs. 2–5(–6)-flowered), standard 14–18(–20) mm, oblong-panduriform, hairy on midline (vs. 20–29(–35) mm, obovate-panduriform, densely appressed hairy on upper side), pods long villous (vs. densely covered with appressed to spreading hairs).

**Figure 5.**
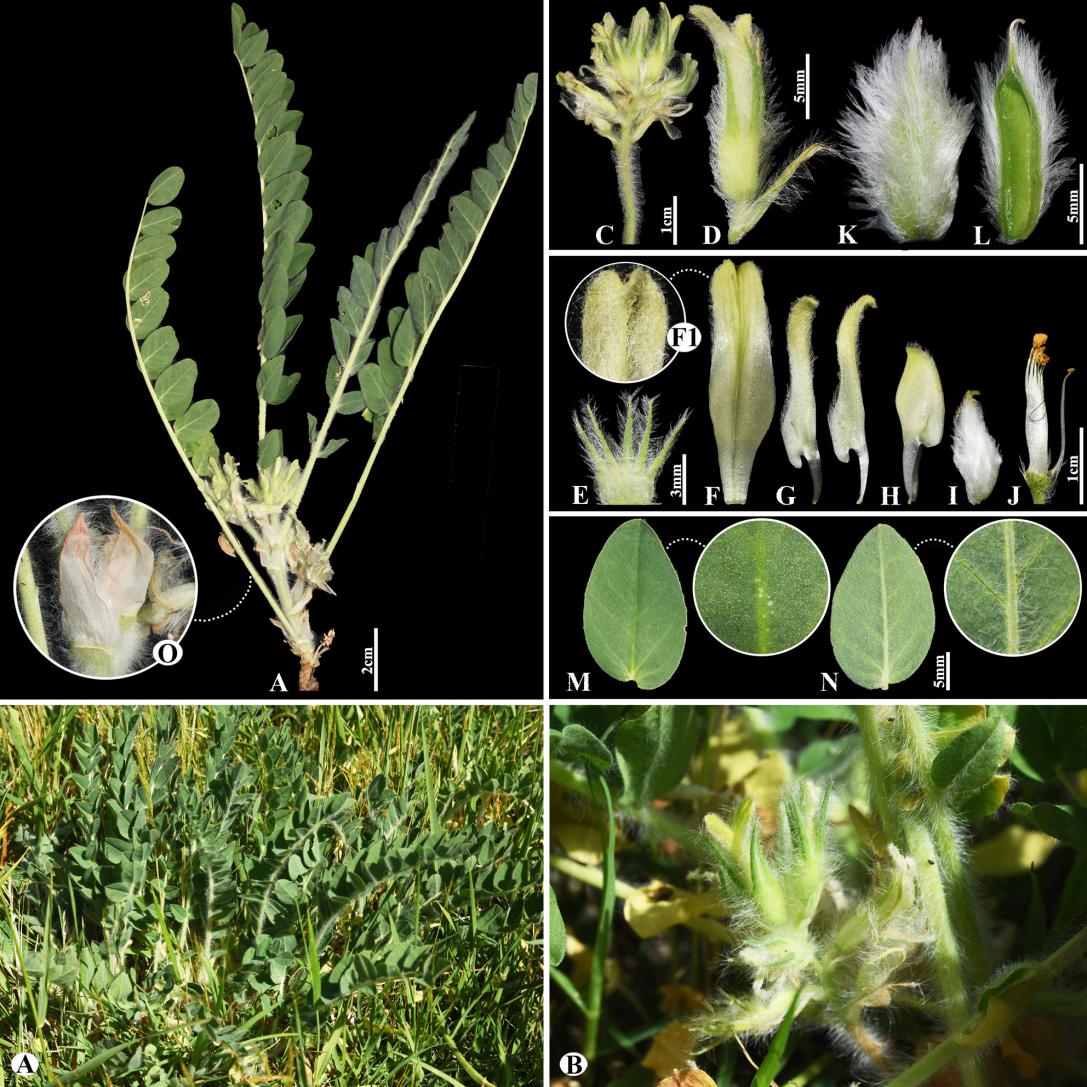
*Astragalus lasiopetalus* in Kazakhstan (Voucher number: MT9). **A**. General habit; **B**. Flowers; **C**. Raceme; **D**. Flower with bract; **E**. Calyx; **F**. Standard; **G**. Wings; **H**. Keel; **I**. Pistil; **J**. Stamens; **K**. Pod; **L**. Pod valve; **M**. Leaflet, adaxial view; **N**. Leaflet, abaxial view; **O**. Stipules. (Photo credits: D.Munkhtulga).

##### Astragalus macronyx

Taxon classification

Plantae

FabalesFabaceae

Bunge, Izv. Imp. Obshch. Lyubit. Estestv. Moskovsk. Univ. 26(2): 236 (1880).

23F85081-E1E3-5C7C-8015-66C2166EC22F

Fig. 6

= Astragalus samarkandinus Freyn, Bull. Herb. Boissier, sér. 2, 4: 763 (1904).

###### Notes.

This species is distributed throughout Afghanistan, Kazakhstan, Kyrgyzstan, Tajikistan and Uzbekistan (POWO 2025). We found this species (voucher number: MT13) on foothill slopes of the Kolsay Lake, Kegen district, occurring at elevations of 1800–1900 m. Morphologically, this species is similar to *A. wolgensis* Bunge but can be distinguished by its leaves (8–)10–25(–32) cm; leaflets 22–29(–32) pairs, narrowly elliptic to elliptic, 15–21 × 8–11 mm (vs. (7–)10–20(–30) cm; leaflets 12–17(–20) pairs, narrowly elliptic to narrowly obovate, 9–14(–18) × 2–5 mm).

**Figure 6.**
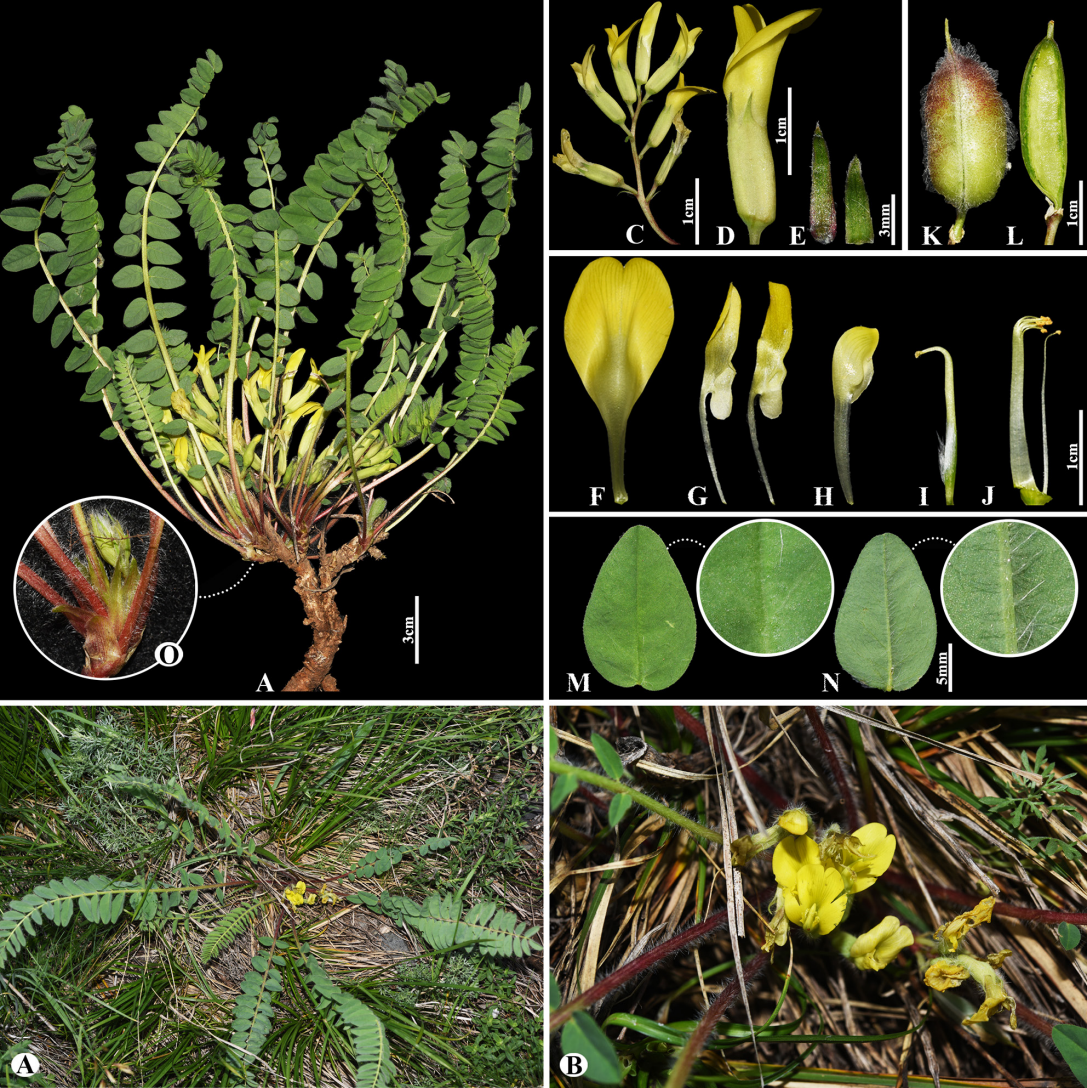
*Astragalus macronyx* in Kazakhstan (Voucher number: MT13). **A**. General habit; **B**. Flowers; **C**. Raceme; **D**. Flower; **E**. Bracts; **F**. Standard; **G**. Wings; **H**. Keel; **I**. Pistil; **J**. Stamens; **K**. Pod; **L**. Pod valve; **M**. Leaflet, adaxial view; **N**. Leaflet, abaxial view; **O**. Stipules. (Photo credits: D.Munkhtulga).

#### Sect. *Erionotus* Bunge, Mém. Acad. Imp. Sci. Saint Pétersbourg 11(16): 32.

##### Astragalus lanuginosus

Taxon classification

Plantae

FabalesFabaceae

Kar. & Kir., Bull. Soc. Imp. Naturalistes Moscou 14: 409 (1841).

2D53E855-BD28-551E-913C-3F6C138D9E04

Fig. 7

≡ Myobroma lanuginosa Steven, Bull. Soc. Imp. Naturalistes Moscou 29(2): 150 (1856).
≡ Tragacantha lanuginosa (Steven) Kuntze, Revis. Gen. Pl. 2: 945 (1891).
= Astragalus anrachaicus Golosk., Bot. Mater. Gerb. Bot. Inst. Komarova Akad. Nauk S.S.S.R. 15: 13 (1953).
= Astragalus larvatus Sumnev., Sist. Zametki Mater. Gerb. Krylova Biol. Inst. Tomsk. Gosud. Univ. Kuybysheva 1936(9–10): 3 (1937).
= Astragalus mucidiformis Sumnev., Sist. Zametki Mater. Gerb. Krylova Tomsk. Gosud. Univ. Kuybysheva 1936(9–10): 1 (1937).
= Astragalus xinjiangnensis Y.C.Ho, Bull. Bot. Res., Harbin 1(3): 121 (1981).

###### Notes.

**Figure 7.**
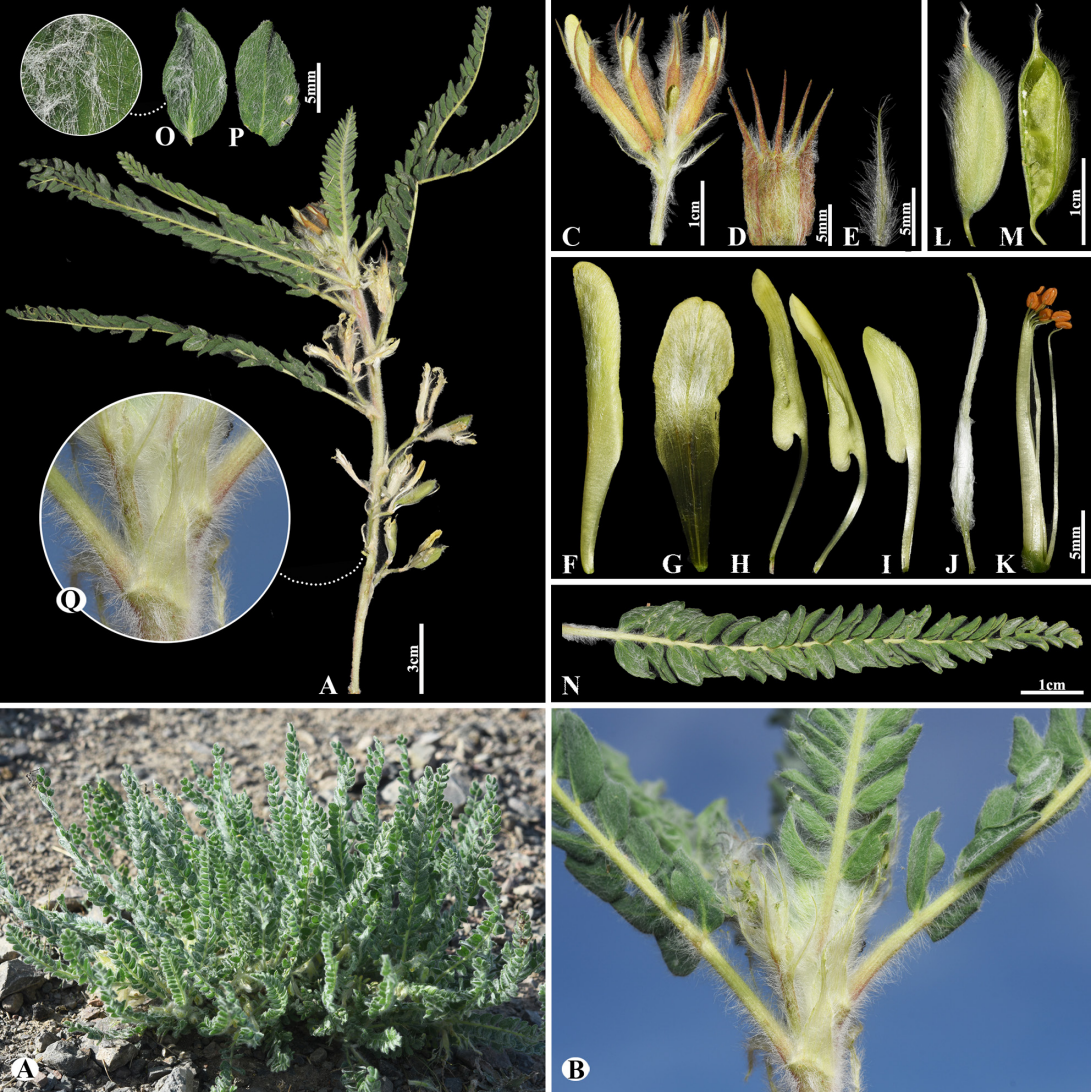
*Astragalus lanuginosus* in Kazakhstan (Voucher number: MT3-1). **A**. General habit; **B**. Leaves; **C**. Raceme; **D**. Calyx; **E**. Bract; **F**. Standard, side view; **G**. Standard, front view; **H**. Wings; **I**. Keel; **J**. Pistil; **K**. Stamens; **L**. Pod; **M**. Pod valve; **N**. Leaf; **O**. Leaflet, adaxial view; **P**. Leaflet, abaxial view; **Q**. Stipules. (Photo credits: D.Munkhtulga).

**Figure 8.**
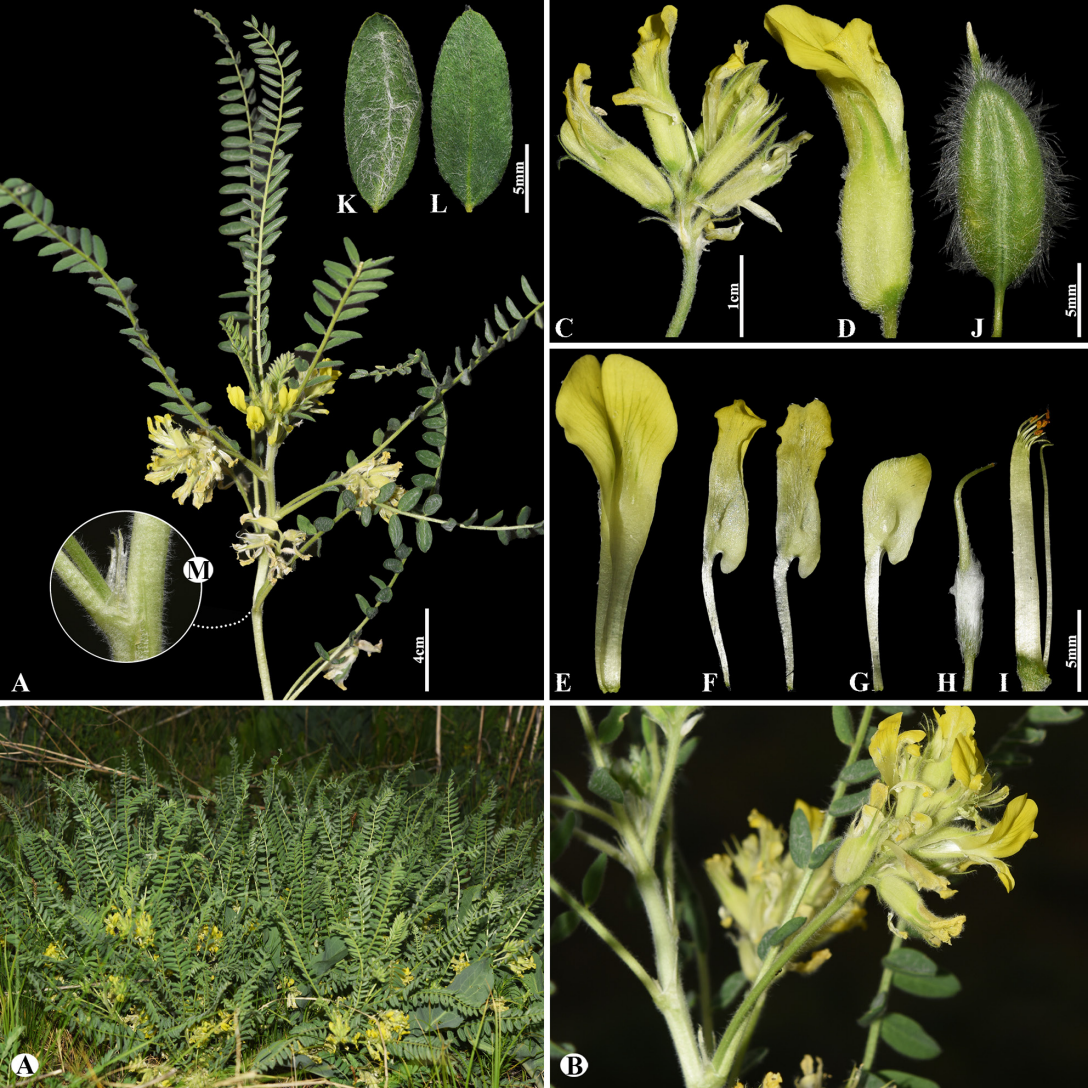
*Astragalus mucidus* in Kazakhstan (Voucher number: MT7-1). **A**. General habit; **B**. Flowers; **C**. Raceme; **D**. Flower; **E**. Standard; **F**. Wings; **G**. Keel; **H**. Pistil; **I**. Stamens; **J**. Pod; **K**. Leaflet, adaxial view; **L**. Leaflet, abaxial view; **M**. Stipules. (Photo credits: D.Munkhtulga).

This species is distributed throughout Kazakhstan, Uzbekistan, Kyrgyzstan, and China (Podlech and Zarre 2013). We found this species (voucher number: MT3-1) in both rocky and rubbly slopes of the Chu-Ily Mountain range, occurring at elevations of 850–900 m. *Astragalus lanuginosus* is related to *A. mucidus* Bunge ex Boiss. (Fig. 8) in inflorescence, calyx and corolla dimensions, and pod of segments, but can be distinguished by leaflets (16–)20–26 pairs, ovate to elliptic or obovate, (4–)5–12 × (3–)4–8 mm, upper side loosely woolly, often glabrescent, underside subappressed hairy (Fig. 7N–P) [vs. 14–26 pairs, narrowly oblong to narrowly ovate, (3–)5–8(–12) × 2–3(–5) mm, upper side cotton wool-like hairy, underside densely hairy (Fig. 8K, L)], bracts 5–10(–12) mm, narrowly triangular, hairy (Fig. 7E) [vs. 4–7 mm, linear-acute, long hairy].

##### Astragalus mucidus

Taxon classification

Plantae

FabalesFabaceae

Bunge ex Boiss., Fl. Orient. 2: 279 (1872).

C68D519D-8286-5DAA-AD36-8DD995C2DEC5

Fig. 8

≡ Myobroma mucida (Bunge ex Boiss.) Nevski, Trudy Bot. Inst. Akad. Nauk S.S.S.R., Ser. 1, Fl. Sist. Vyssh. Rast. 4: 256 (1937).
≡ Tragacantha mucida (Bunge ex Boiss.) Kuntze, Revis. Gen. Pl. 2: 946 (1891).
= Astragalus mucifer Bunge, Izv. Imp. Obshch. Lyubit. Estestv. Moskovsk. Univ. 26(2): 223 (1880).
= Astragalus serafichanicus Freyn, Bull. Herb. Boissier, sér. 2, 4: 767 (1904).

###### Notes.

This species is distributed throughout Kazakhstan, Tajikistan, Uzbekistan, and Kyrgyzstan (Podlech and Zarre 2013). We found this species (voucher number: MT7-1) in rocky and rubbly slopes, abandoned fields of the Kurday, occurring at elevations of 1,200–1,300 m. *Astragalus mucidus* is morphologically similar to *A. floccosifolius* Sumnev., but can be distinguished by its calyx densely covered with curly hairs (vs. with ascending to spreading hairs), standard 14–18 mm long (vs. (17–)18–24 mm long).

#### *Astragalus* subgenus *Calycophysa* Bunge, Astrag. geront. 56 (2): 95 (1868).

**Sect. *Alopecuroidei* DC., Prodr. 2: 294 (1825)**.

##### Astragalus vulpinus

Taxon classification

Plantae

FabalesFabaceae

Willd., Sp. Pl., ed. 4. 3: 1259 (1802).

2300EDB4-B9C7-51FF-B7FD-58B269C8A574

Fig. 9

≡ Alopecias vulpinus (Willd.) Steven, Bull. Soc. Imp. Naturalistes Moscou 29(2): 143 (1856).
= Astragalus lagocephalus Fisch. & C.A.Mey. ex Schrenk, Bull. Cl. Phys.-Math. Acad. Imp. Sci. Saint-Pétersbourg 2: 197 (1844).
= Tragacantha lagocephala (Fisch. & C.A.Mey. ex Schrenk) Kuntze, Revis. Gen. Pl. 2: 945 (1891).

###### Notes.

This species is distributed in eastern and southern European Russia, western Siberia, Kazakhstan, Uzbekistan, and China (Xinjiang) (Podlech and Zarre 2013). We found this species (voucher number: MT6-2) in clayey and rocky sites of the Chu-Ily Mountain range, occurring at elevations of 800–850 m. *Astragalus vulpinus* is closely related to *A. alopecurus* Pall. but differs in its inflorescence on short or long peduncles, ovate or ovateoblong, 4–6 cm long (sessile or subsessile, oval or cylindrical, 5–15 cm long), and leaflets 12–15 pairs (vs. 15–25 (–30) pairs).

**Figure 9.**
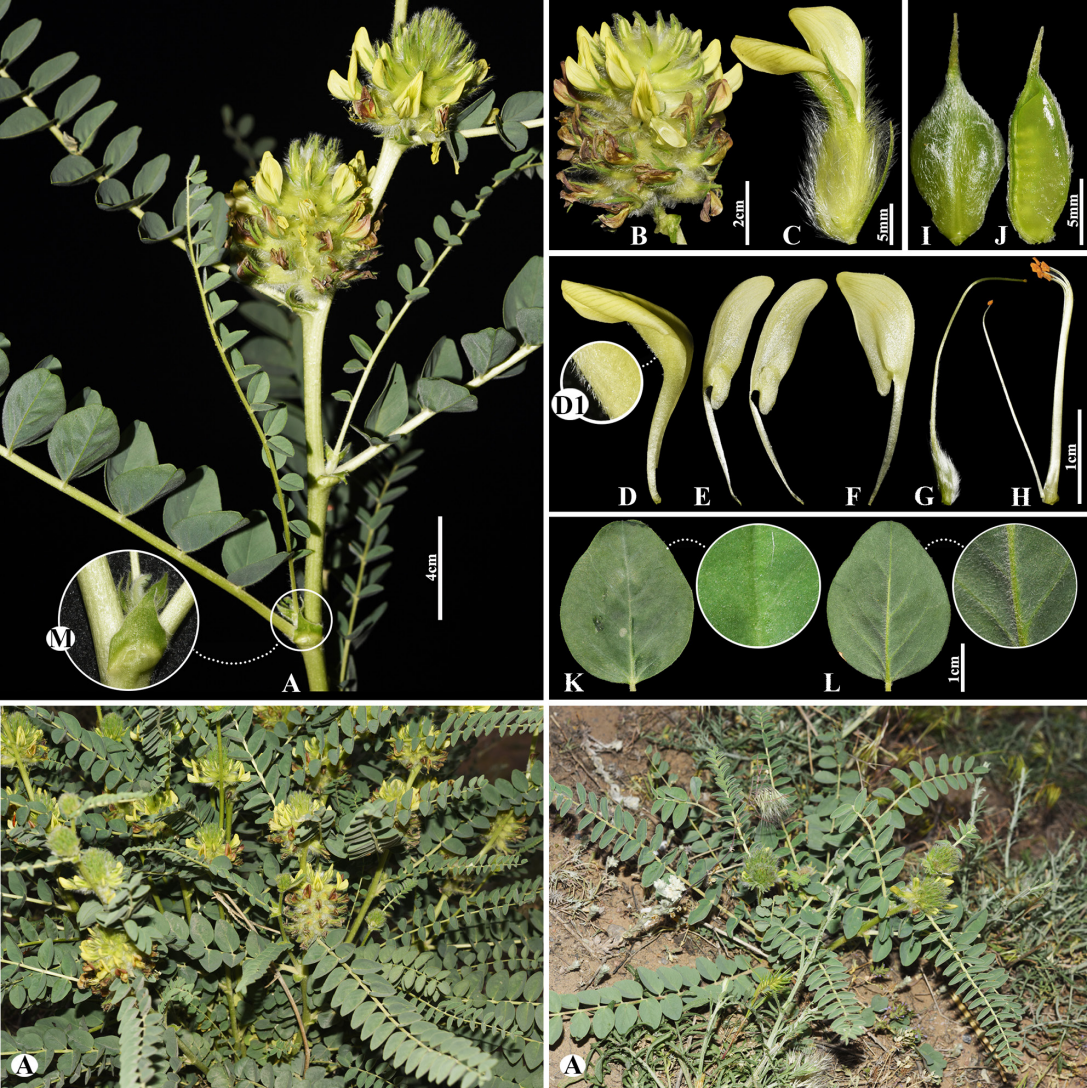
*Astragalus vulpinus* in Kazakhstan (Voucher number: MT6-2). **A**. General habit; **B**. Raceme; **C**. Flower; **D**. Standard; **D1**. Standard hairs; **E**. Wings; **F**. Keel; **G**. Pistil; **H**. Stamens; **I**. Pod; **J**. Pod valve; **K**. Leaflet, adaxial view; **L**. Leaflet, abaxial view; **M**. Stipules. (Photo credits: D.Munkhtulga).

#### Sect. *Eremophysa* Bunge, Mém. Acad. Imp. Sci. Saint Pétersbourg 11(16): 62 (1868).

##### Astragalus sphaerophysa

Taxon classification

Plantae

FabalesFabaceae

Kar. & Kir., Bull. Soc. Imp. Naturalistes Moscou 15: 338 (1842).

8A7725E8-2F61-583D-9CFF-1C40D4371421

Fig. 10

≡ Tragacantha sphaerophysa (Kar. & Kir.) Kuntze, Revis. Gen. Pl. 2: 948 (1891).

###### Notes.

This species occurs in Kazakhstan and northwestern China and is restricted to Central Asia (Podlech and Zarre 2013). We found this species (voucher number: MT5-12) in the fixed sand of the Chu-Ily Mountain range, occurring at elevations of 400–450 m. *Astragalus sphaerophysa* is morphologically similar to *A. citoinflatus* Bondarenko, but can be distinguished by its calyx at anthesis 12–14 mm, its teeth c. 1/2 of the tube length; fruiting calyx 15–20 mm (vs. calyx at anthesis 10–12 mm, its teeth c. 2/3 of the tube length; fruiting calyx 15–16 mm), pods with a stipe 3–4 mm (vs. with a stipe c. 2 mm).

**Figure 10.**
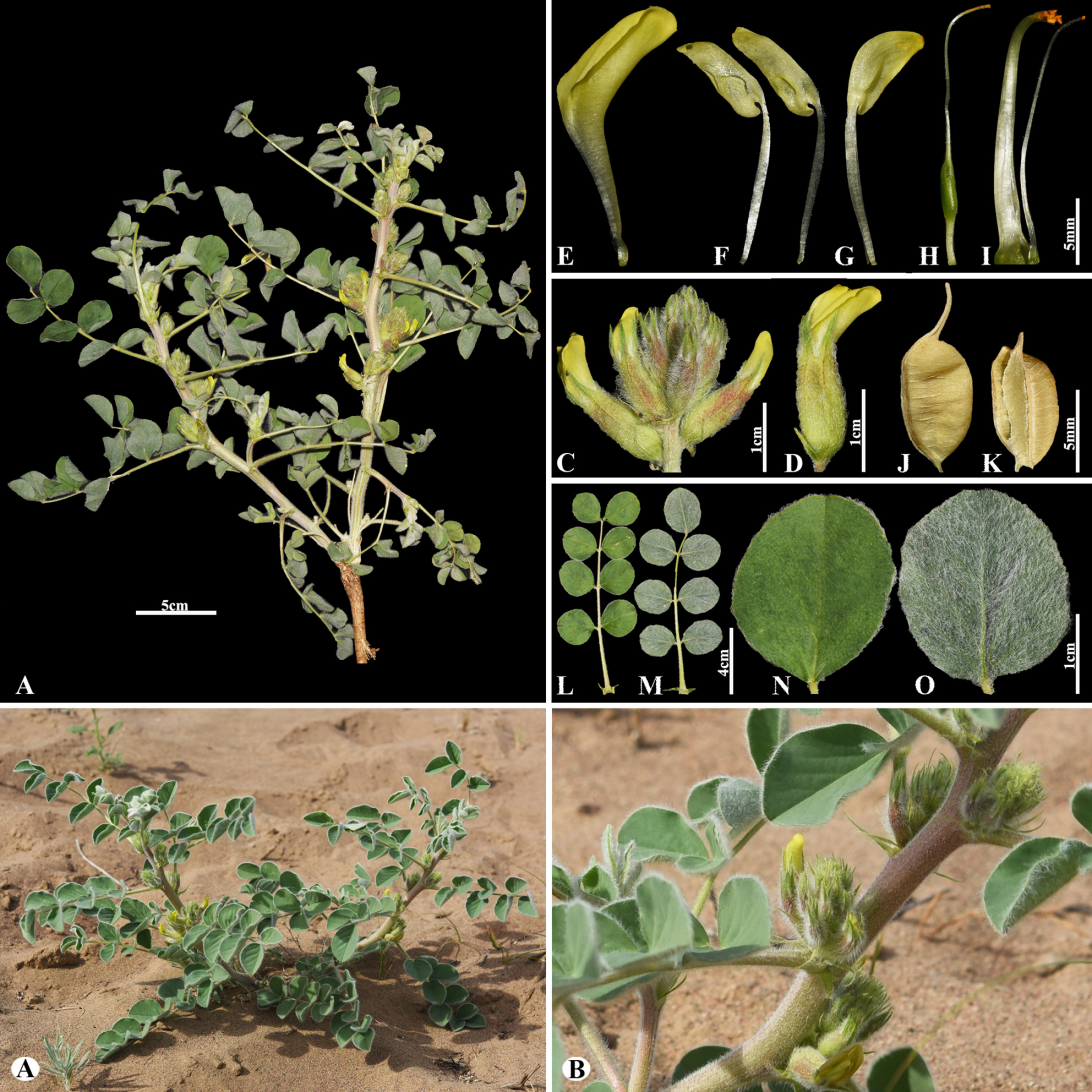
*Astragalus sphaerophysa* in Kazakhstan (Voucher number: MT5-12). **A**. General habit; **B**. Flowering branch; **C**. Raceme; **D**. Flower; **E**. Standard; **F**. Wings; **G**. Keel; **H**. Pistil; **I**. Stamens; **J**. Pod; **K**. Pod valve; **L**. Leaf, adaxial view; **M**. Leaf, abaxial view; **N**. Leaflet, adaxial view; **O**. Leaflet, abaxial view. (Photo credits: D.Munkhtulga).

#### Sect. *Lithoon* (Nevski) Gontsch., Fl. URSS 12: 98 (1946).

##### Astragalus sieversianus

Taxon classification

Plantae

FabalesFabaceae

Pall., Sp. Astragal.: 15 (1800).

FDB157FE-A4C4-56AB-838C-E9362EF1B641

Fig. 11

≡ Alopecias sieversianus (Pall.) Steven, Bull. Soc. Imp. Naturalistes Moscou 29(2): 143 (1856).
≡ Lithoon sieversianum (Pall.) Nevski, Trudy Bot. Inst. Akad. Nauk S.S.S.R., Ser. 1, Fl. Sist. Vyssh. Rast. 4: 255 (1937).
≡ Tragacantha sieversiana (Pall.) Kuntze, Revis. Gen. Pl. 2: 948 (1891).
= Astragalus christianus Siev., Neueste Nord. Beytr. Phys. Geogr. Erd- Völkerbeschreib. 7: 293 (1796), nom. illeg.

###### Notes.

*Astragalus sieversianus* belongs to the sect. *Lithoon*, which is an ancient monotypic section. It was described from Ul’dzhar in Kazakhstan and is distributed in Afghanistan, Iran, Kazakhstan, Kyrgyzstan, Tajikistan, Turkmenistan, Uzbekistan, and China (Xinjiang) (Podlech and Zarre 2013). We found this species (voucher number: MT7-5) in foothills and mountain slopes of the Kurday, Korday district, occurring at elevations of 1,200–1,300 m.

**Figure 11.**
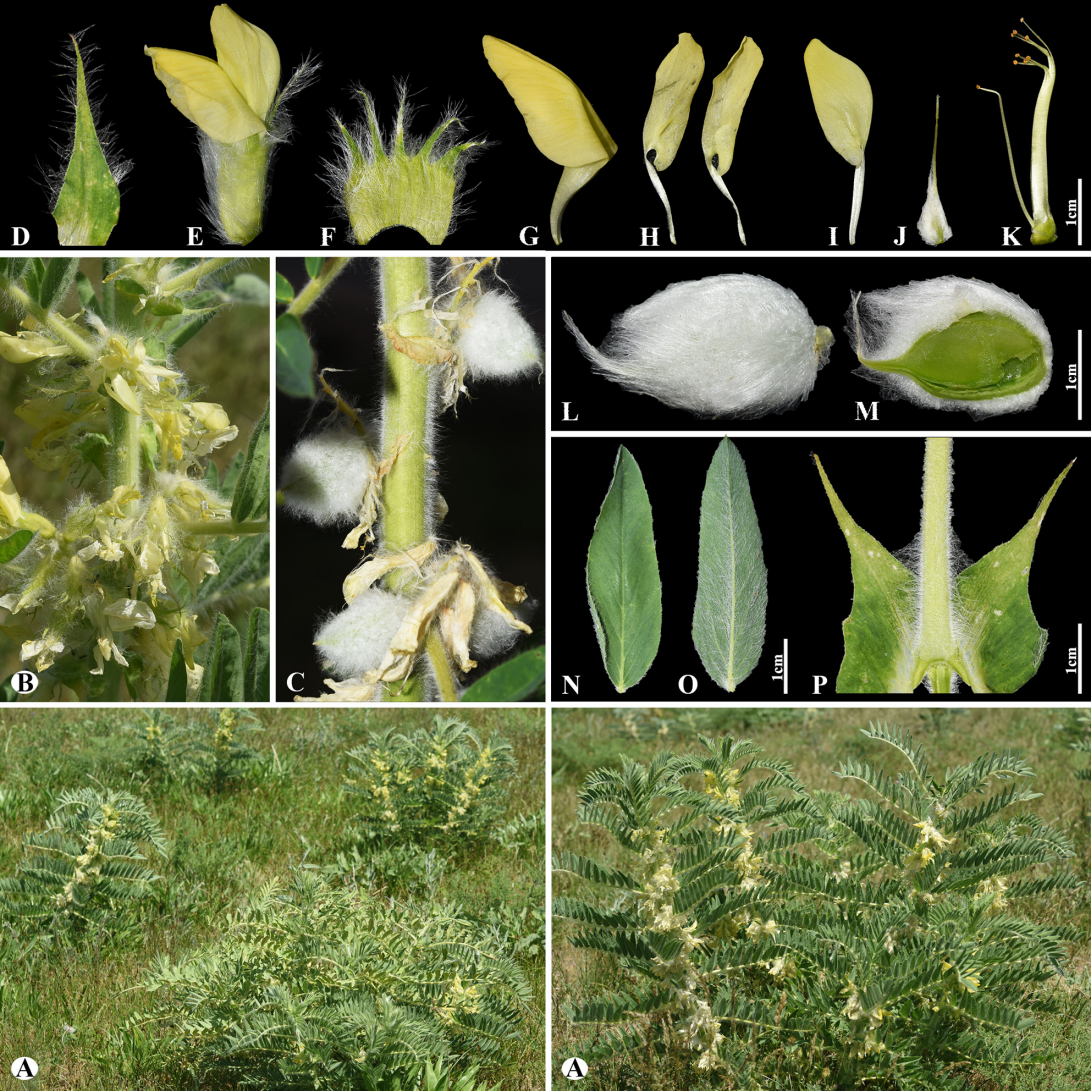
*Astragalus sieversianus* in Kazakhstan (Voucher number: MT7-5). **A**. General habits; **B**. Raceme; **C**. Pods; **D**. Bracts; **E**. Flower; **F**. Calyx; **G**. Standard; **H**. Wings; **I**. Keel; **J**. Pistil; **K**. Stamens; **L**. Pod; **M**. Pod valve; **N**. Leaflet, adaxial view; **O**. Leaflet, abaxial view; **P**. Stipules. (Photo credits: D.Munkhtulga).

#### *Astragalus* subgenus *Cercidothrix* Mem. Acad. Imp. Sci. Saint Petersburg, 11, 16: 94 (1868).

**Sect. *Ammodendron* Bunge, Mém. Acad. Imp. Sci. Saint Pétersbourg 11(16): 128 (1868)**.

##### Astragalus brachypus

Taxon classification

Plantae

FabalesFabaceae

Schrenk ex Fisch. & C.A.Mey., Enum. Pl. Nov. 1: 79 (1841).

D99047DF-366A-5B46-94CB-5A097CC4BE5F

Fig. 12

≡ Solenotus brachypus (Schrenk) Steven, Bull. Soc. Imp. Naturalistes Moscou 29(2): 144 (1856).
≡ Tragacantha brachypus (Schrenk) Kuntze, Revis. Gen. Pl. 2: 943 (1891).
= Astragalus halodendron Bunge, Bull. Soc. Imp. Naturalistes Moscou 39(2): 21 (1866).
= Tragacantha halodendron (Bunge) Kuntze, Revis. Gen. Pl. 2: 945 (1891).

###### Notes.

This species was found in Kazakhstan and the NE. China (Podlech and Zarre 2013). We found this species (voucher number: MT5-3) in semi-fixed dune sand of the Chu-Ily Mountain range, occurring at elevations of 400–450 m. *Astragalus brachypus* is morphologically similar to *A. hotianensis* S.B.Ho, but can be distinguished by its calyx 6–7 mm long (vs. 4–6 mm long), pods oblong, 5–8 mm long, with densely villous (vs. 1–3 pairs, filiform-linear, not more than 1 mm broad).

**Figure 12.**
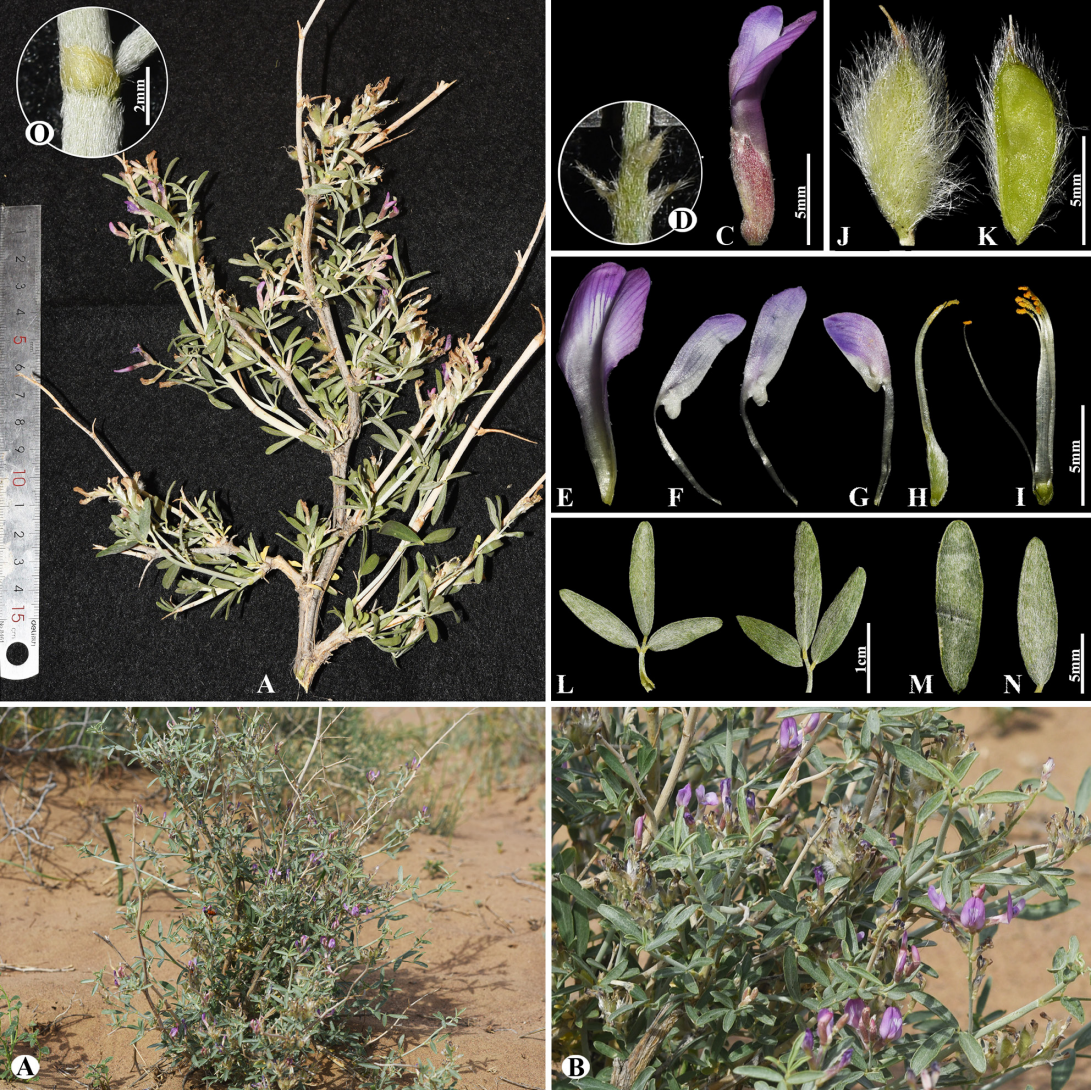
*Astragalus brachypus* in Kazakhstan (Voucher number: MT5-3). **A**. General habit; **B**. Flowering branch; **C**. Flower; **D**. Bracts; **E**. Standard; **F**. Wings; **G**. Keel; **H**. Pistil; **I**. Stamens; **J**. Pod; **K**. Pod valve; **L**. Leaf; **M**. Leaflet, adaxial view; **N**. Leaflet, abaxial view; **O**. Stipules. (Photo credits: D.Munkhtulga).

##### Astragalus paucijugus

Taxon classification

Plantae

FabalesFabaceae

Schrenk, Bull. Cl. Phys.-Math. Acad. Imp. Sci. Saint-Pétersbourg 2: 196 (1844).

8592063E-1560-5D81-8A0B-218216F12A89

Fig. 13

≡ Astragalus ammodendron subsp. paucijugus (Schrenk) Basil. in Trudy Prikl. Bot. 18: 546 (1928).
≡ Tragacantha paucijuga (Schrenk) Kuntze, Revis. Gen. Pl. 2: 947 (1891).
= Astragalus arborescens Bunge, Arbeiten Naturf. Vereins Riga 1: 229 (1847).

###### Notes.

This species is distributed throughout Kazakhstan, Turkmenistan and Uzbekistan (Podlech and Zarre 2013). We found this species (voucher number: MT5-10) in semi-fixed dune sand of the Chu-Ily Mountain range, occurring at elevations of 400–450 m. *Astragalus paucijugus* is morphologically similar to *A. ammodendron* Bunge, but can be distinguished by its leaflets 1–3 pairs, elliptic to obovate, 8–30 × 3–12 mm, upper surface glabrous, lower surface sparsely hairy (vs. 3–5 pairs, linear, lanceolate or oblong, 1 mm or more broad), pods 8–12 × 3–3.5 mm, beak c. 1 mm, bilocular, valves thin but tough, densely covered with long basifixed white hairs (vs. 7–11 × ~3 mm, beak 3–4 mm, bilocular, valves rigidly membranous, densely covered with spreading hairs on small tubercles).

**Figure 13.**
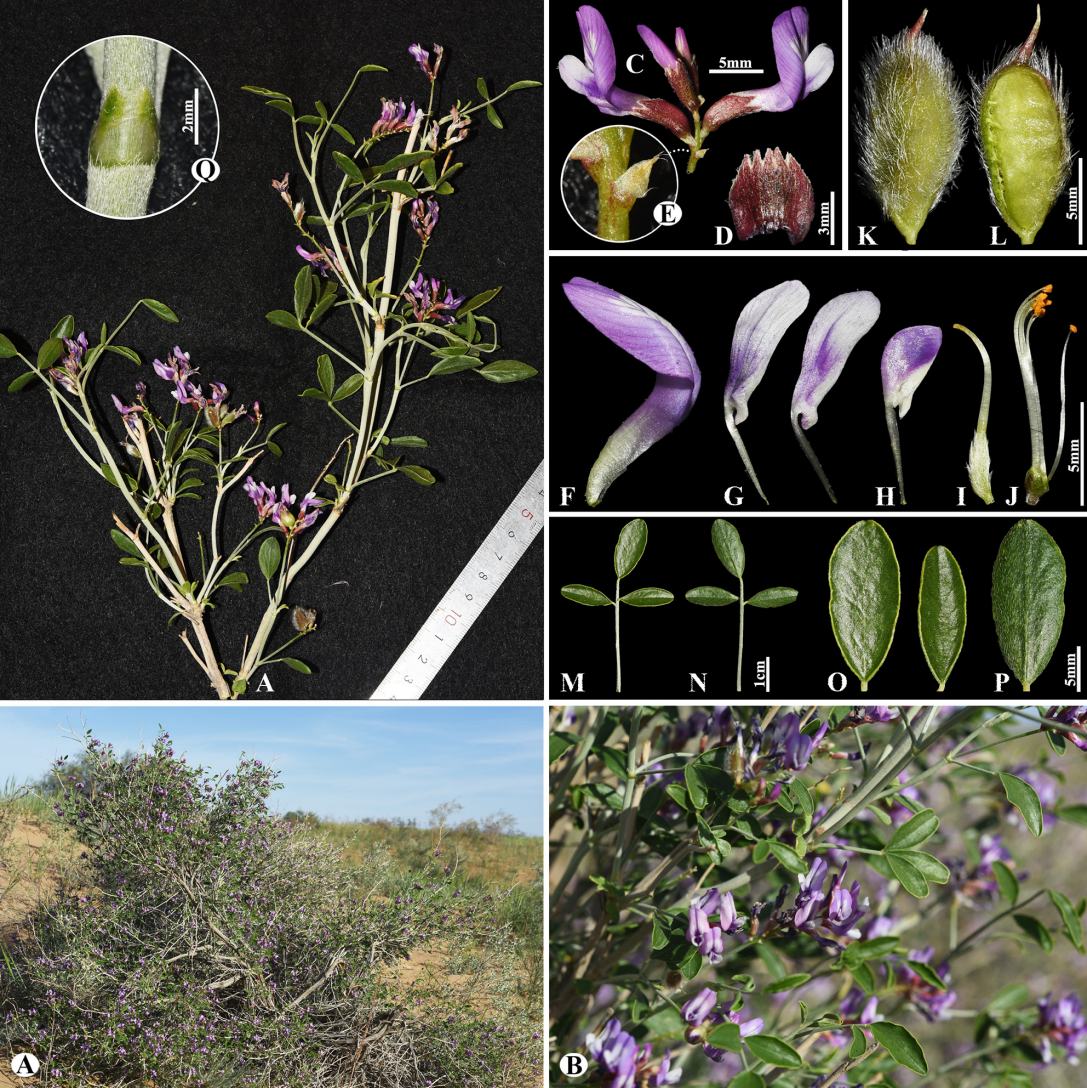
*Astragalus paucijugus* in Kazakhstan (Voucher number: MT5-10). **A**. General habit; **B**. Flowering branch; **C**. Raceme; **D**. Calyx; **E**. Bract; **F**. Standard; **G**. Wings; **H**. Keel; **I**. Pistil; **J**. Stamens; **K**. Pod; **L**. Pod valve; **M**. Leaf, adaxial view; **N**. Leaf, abaxial view; **O**. Leaflet, adaxial view; **P**. Leaflet, abaxial view; **Q**. Stipules. (Photo credits: D.Munkhtulga).

#### Sect. *Chaetodon* Bunge, Mém. Acad. Imp. Sci. Saint Pétersbourg 11(16): 136 (1868).

##### Astragalus chaetodon

Taxon classification

Plantae

FabalesFabaceae

Bunge, Beitr. Fl. Russl.: 96 (1852).

C4A1FB17-36AE-5969-BB49-3467D0959AD0

Fig. 14

≡ Tragacantha chaetodon (Bunge) Kuntze, Revis. Gen. Pl. 2: 943 (1891).

###### Notes.

**Figure 14.**
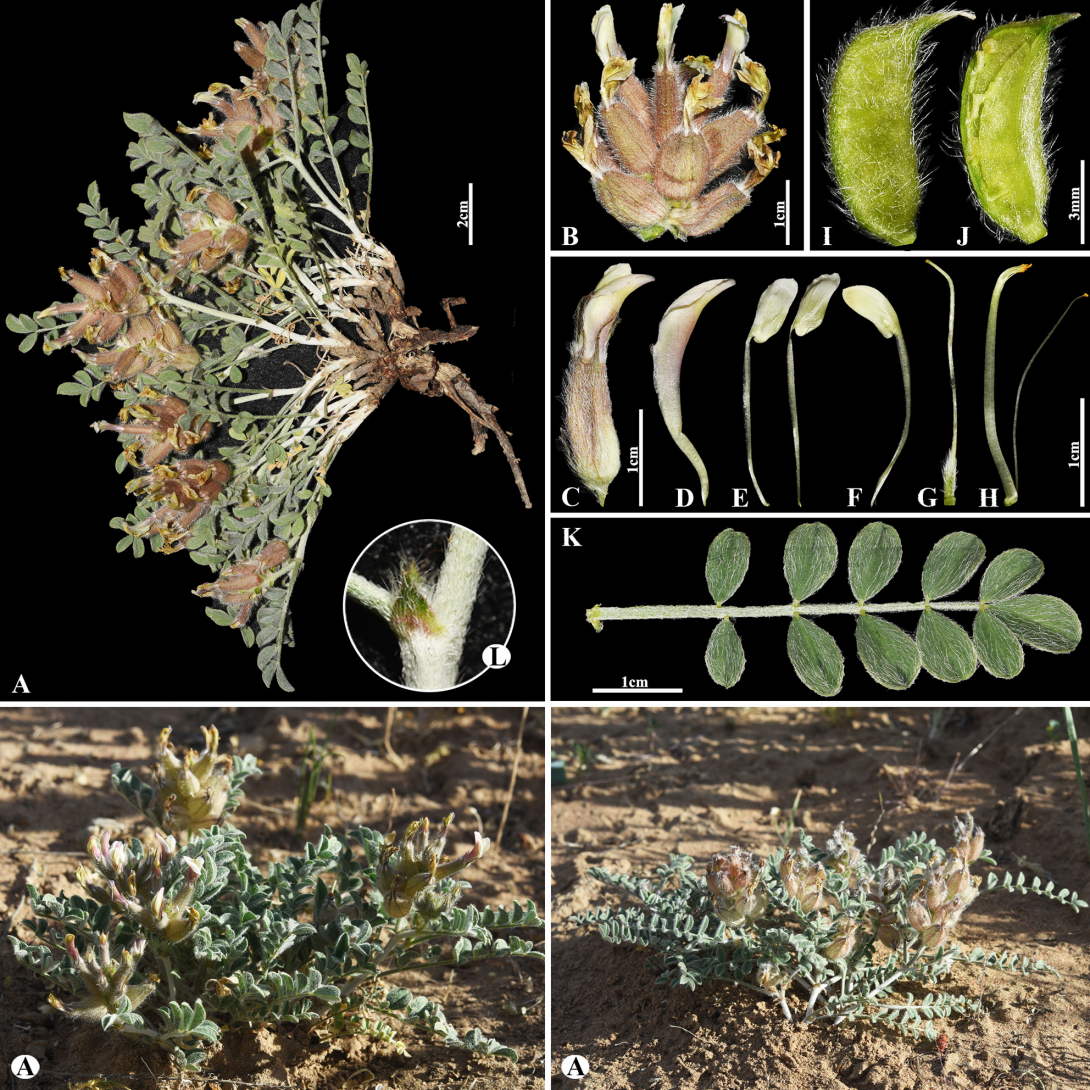
*Astragalus chaetodon* in Kazakhstan (Voucher number: MT5-6). **A**. General habit; **B**. Raceme; **C**. Flower; **D**. Standard; **E**. Wings; **F**. Keel; **G**. Pistil; **H**. Stamens; **I**. Pod; **J**. Pod valve; **K**. Leaf; **L**. Stipules. (Photo credits: D.Munkhtulga).

This species is distributed throughout Kazakhstan and Uzbekistan (Podlech and Zarre 2013). We found this species (voucher number: MT5-6) in the clayey and sandy soils of the submontane plains and foothills of the Kanshengel, Zhambyl district, occurring at elevations of 400–420 m. *Astragalus chaetodon* closely resembles *A. psilolobus* Puchkova (Fig. 16) in leaf shape and general habit, but differs in its height (5–)10–20(–40) cm tall, several stems, 2–12(–35) cm, angular, branched at base (Fig. 14A) [vs. 5–8 cm, subacaulescent or nearly stemless, very short stems (Fig. 16A)], leaflet (5–)6–9 pairs, elliptic–obovate, 4–12(–15) × 2–5 mm (Fig. 14K) [vs. (4–)5–6 pairs, ovate, 5–10 × 2–5 mm (Fig. 16G)], petals pale pinkish violet to yellowish (Fig. 14C–F) [vs. purple or violet (Fig. 16D)], pods sessile, ellipsoid, 5–9 × 3–4.5 mm, bilocular, curved beak 1–1.5 mm, densely spreading hairs (Fig. 14I–J) [vs. sessile, oblong, 11–15 × 2–3 mm, incompletely bilocular, short curved beak, glabrous or with scattered basifixed spreading hairs (Fig. 16E–F)].

**Figure 15.**
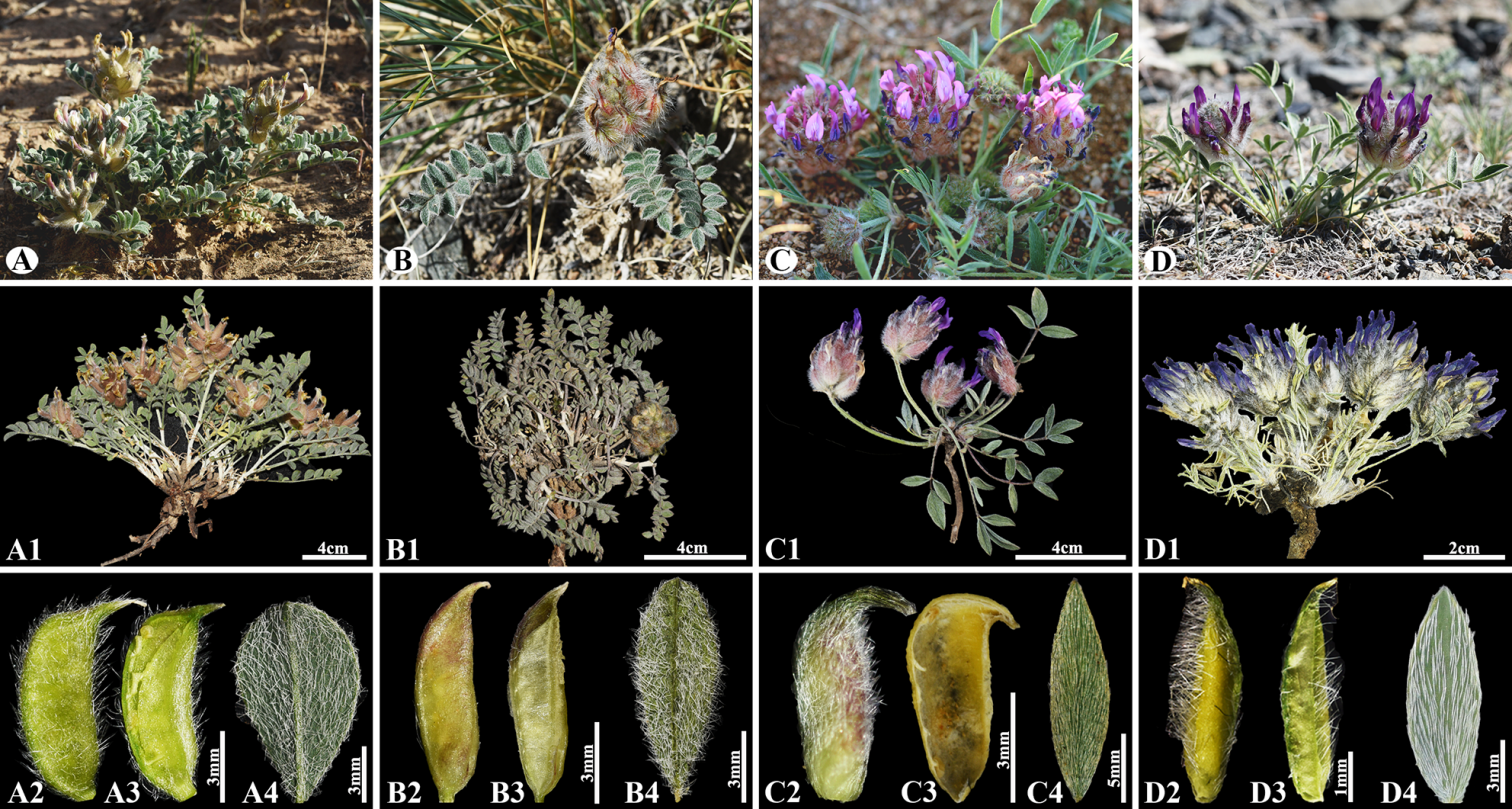
General habit and morphological characteristics of pods and leaflets in selected *Astragalus* species. **A–A4**. *A. chaetodon* (sect. *Chaetodon*) (Voucher Number: MT5-6); **B–B4**. *A. psilolobus* (sect. *Chaetodon*) (Voucher number: MT4-3); **C–C4**. *A. laguroides* (sect. *Laguropsis*) (Voucher number: West24-1); **D–D4**. *A. uvsicus* (sect. *Uvsicus*) (Voucher number: West24-5). 1. Habits; 2. Pods; 3. Pod valves; 4. Leaflets. (Photo credits: D.Munkhtulga).

**Figure 16.**
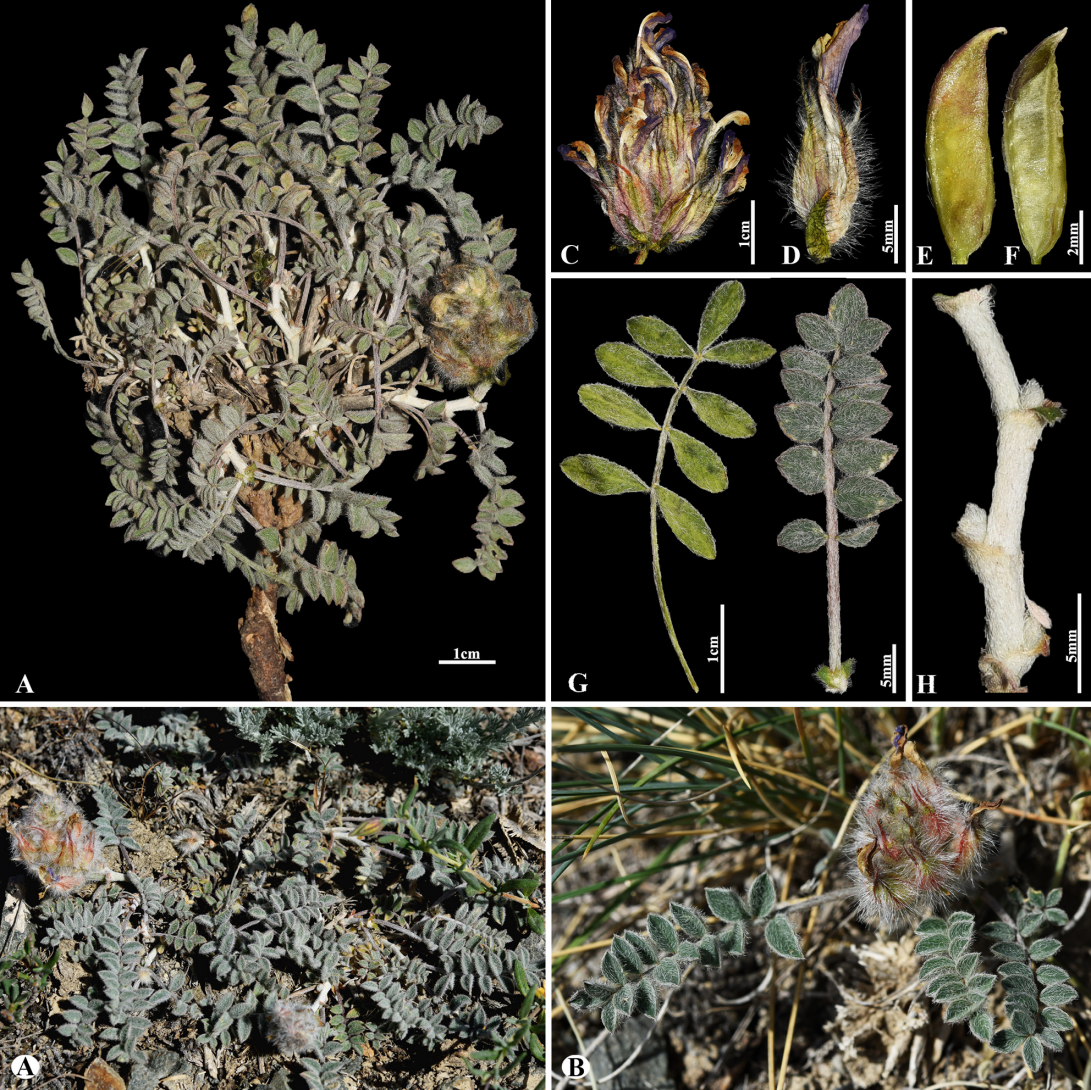
*Astragalus psilolobus* in Kazakhstan (Voucher number: MT4-3). **A**. General habit; **B**. Flowering branch; **C**. Raceme; **D**. Flower; **E**. Pod; **F**. Pod valve; **G**. Leaves; **H**. Stem. (Photo credits: D.Munkhtulga).

##### Astragalus psilolobus

Taxon classification

Plantae

FabalesFabaceae

Puchkova, Vidy Sekts. Chaetodon v Astrag.: 48 (1967).

648AD9B5-7F69-5310-9F58-C1131A05EBCB

Fig. 16

###### Notes.

This species is endemic and was originally described in Kyzyl-Zhar, located north of Taldy-Kurgan in Kazakhstan (Puchkova 1967). We found this species (voucher number: MT4-3) on the rocky, rubbly, steppified slopes of the foothills of the Chu-Ily Mountain, occurring at elevations of 1,100–1,150 m. Although the species was originally described in the sect. *Chaetodon*, it has subsequently been treated within the sect. *Laguropsis* in major taxonomic studies on *Astragalus* (Ulziykhutag 2003; Podlech and Zarre 2013). In general, it is very similar to *A. laguroides* but can be easily distinguished by its short stems, leaflets densely covered with spreading hairs. According to previous literature (Puchkova 1967; Podlech and Zarre 2013), the taxonomic status of *A. chaetodon* and *A. psilolobus* from sect. *Chaetodon* is still doubtful. In addition, many photo observations of both species have been uploaded to the iNaturalist and Plantarium platforms under the *A. chaetodon* name. In the past, Puchkova (1967) has revised sect. *Chaetodon* based on morphological evidence, and described *A. psilolobus* from the Chu-Ili Mountains in Kazakhstan. According to Puchkova (1967), sect. *Chaetodon* was divided into four groups, with *A. psilolobus* being a separate group, emphasizing that the sectional position of this species should be determined based on phylogenetic analysis and extensive morphological studies. Subsequently, *A. psilolobus* was transferred to sect. *Laguropsis* from sect. *Chaetodon* is based only on its description (Podlech and Zarre 2013). Therefore, in the present study, we identified both species based on their morphological and phylogenetic data. Based on our phylogenetic results, *A. psilolobus* is closely associated with *A. arbuscula* (sect. *Dissitiflori*), rather than the species of sect. *Chaetodon*. In addition, our morphological studies confirmed that *A. psilolobus* is different from *A. laguroides* (Fig. 15C; type species of sect. *Laguropsis*) and *A. uvsicus* Munkht. & Baasanm. (Fig. 15D; type species of sect. *Uvsicus* from Mongolia) by its short stems, leaflets densely covered with spreading hair (Fig. 15).

#### Sect. *Corethrum* Bunge, Mém. Acad. Imp. Sci. Saint Pétersbourg 11(16): 98 (1868).

##### Astragalus kronenburgii

Taxon classification

Plantae

FabalesFabaceae

B.Fedtsch. & Kneuck., Allg. Bot. Z. Syst. 11: 171 (1905).

2FEE5C41-0FAB-5964-899C-E9C8E831D754

Fig. 17

= Astragalus kujukensis B.Fedtsch., Trudy Imp. S.-Peterburgsk. Bot. Sada 24: 225 (1905).

###### Notes.

This species is endemic to Central Asia and occurs in Kazakhstan, Kyrgyzstan, and Uzbekistan (Podlech and Zarre 2013). We found this species (voucher number: MT2-1) on the rocky and rubbly slopes of the Chu-Ily Mountain, occurring at elevations of 800–900 m. *Astragalus kronenburgii* is morphologically similar to *A. nematodes* Bunge ex Boiss., but can be distinguished by its leaflets 3–5 pairs, linear, lanceolate or oblong, 1 mm or more broader (vs. 1–3 pairs, filiform-linear, not more than 1 mm broad).

**Figure 17.**
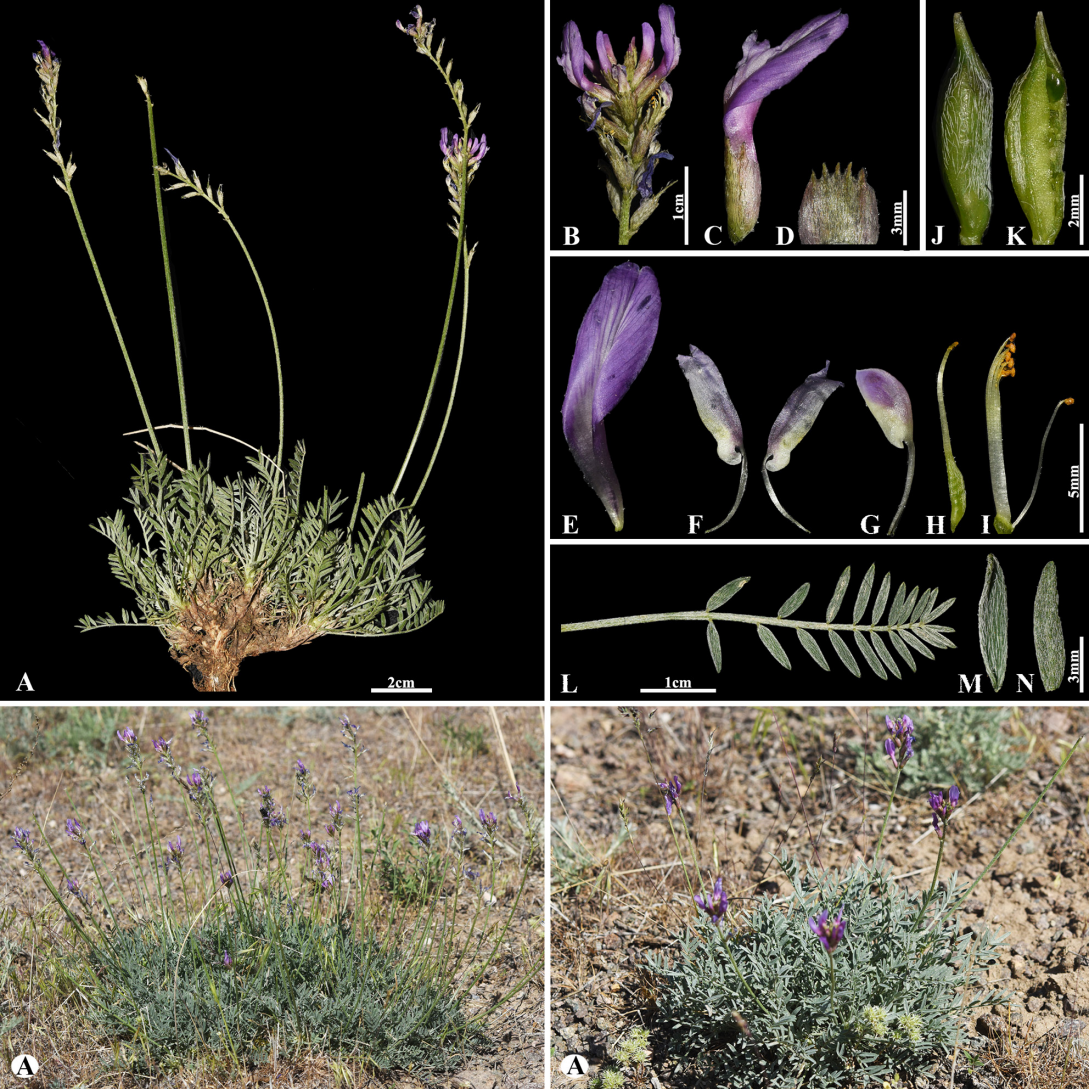
*Astragalus kronenburgii* in Kazakhstan (Voucher number: MT2-1). **A**. General habit; **B**. Raceme; **C**. Flower; **D**. Calyx; **E**. Standard; **F**. Wings; **G**. Keel; **H**. Pistil; **I**. Stamens; **J**. Pod; **K**. Pod valve; **L**. Leaf; **M**. Leaflet, adaxial view; **N**. Leaflet, abaxial view. (Photo credits: D.Munkhtulga).

#### Sect. *Cytisodes* Bunge, Mém. Acad. Imp. Sci. Saint Pétersbourg 11 (16): 127 (1868).

##### Astragalus pseudocytisoides

Taxon classification

Plantae

FabalesFabaceae

Popov, Bot. Mater. Gerb. Bot. Inst. Komarova Akad. Nauk S.S.S.R. 10: 18 (1947).

BC73F469-C72F-55B1-B7DE-EFE64887A06D

Fig. 18

= Astragalus krassnovianus Gontsch., Bot. Mater. Gerb. Bot. Inst. Komarova Akad. Nauk S.S.S.R. 10: 40 (1947).

###### Notes.

This species is a narrowly endemic taxon restricted to the Czu-Ili Mountains between Ak-Taschet and Chan-tau in Kazakhstan (Kubentayev et al. 2024). We found this species (voucher number: MT4-2) in stony mountain slopes of the Chu-Ily Mountain, occurring at elevations of 1,100–1,200 m. The morphological characters of this species are very similar to *A. cytisoides* Bunge, but it can be distinguished by its pods sessile, narrowly oblong 20–30 mm long, slightly compressed laterally, obtusely keeled ventrally and dorsally, beak 12–30 mm, bilocular; valves coriaceous, densely villous with basifixed, spreading, tangled white hairs up to 4 mm (vs. subsessile, linear-oblong 19–35 × 5–6 mm, compressed laterally, not grooved dorsally, beak ~5 mm, bilocular; valves densely villous with subbasifixed white hairs 1.2–1.5 mm).

**Figure 18.**
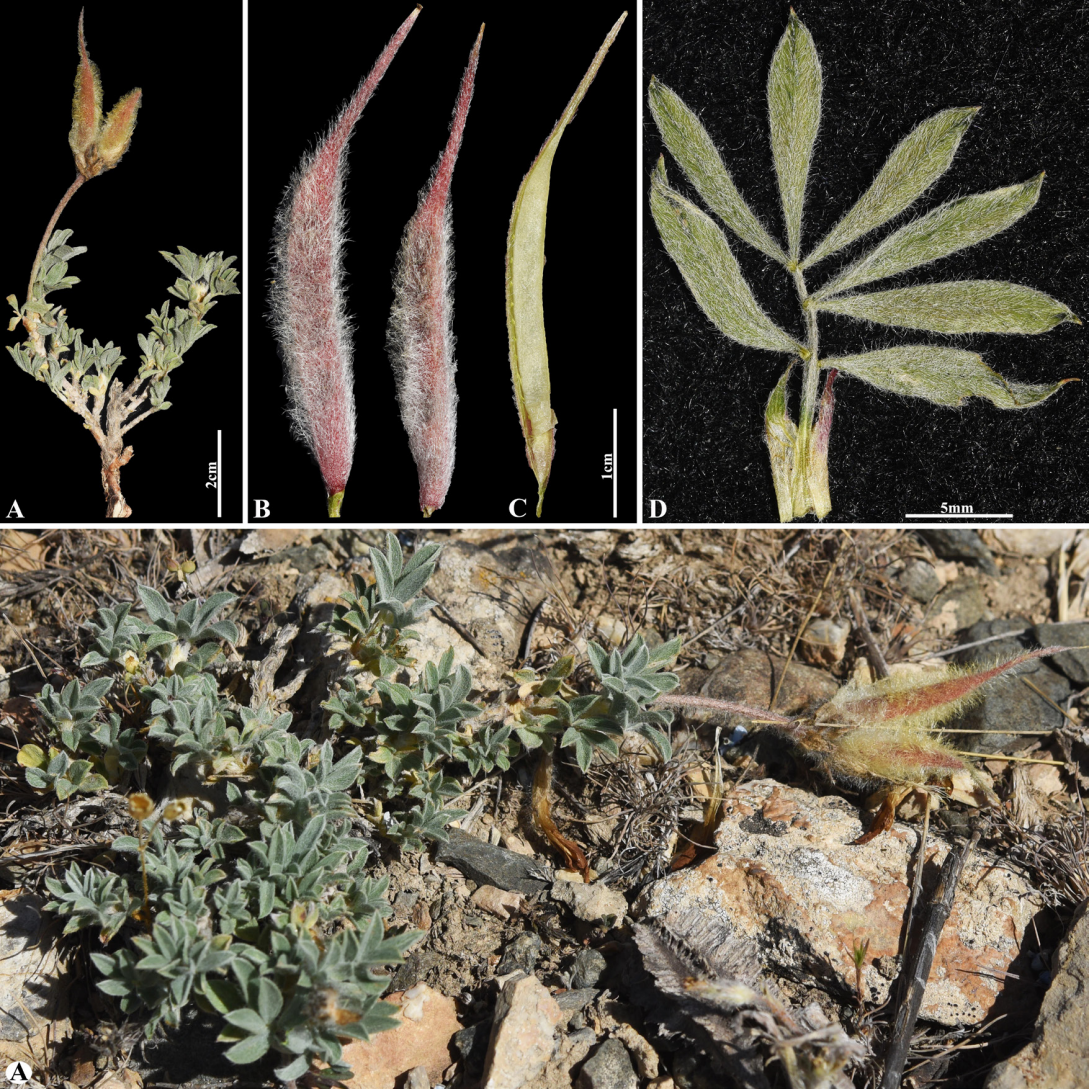
*Astragalus pseudocytisoides* in Kazakhstan (Voucher number: MT4-2). **A**. General habit; **B**. Pod; **C**. Pod valve; **D**. Leaf. (Photo credits: D.Munkhtulga).

#### Sect. *Dissitiflori* DC., Prodr. 2: 284 (1825).

##### Astragalus arbuscula

Taxon classification

Plantae

FabalesFabaceae

Pall., Sp. Astragal.: 24 (1800).

A01BFA5E-8604-5421-AE0E-85DC5151D403

Fig. 19

≡ Astragalus arbuscula var. *mixotricha* Trautv., Bull. Soc. Imp. Naturalistes Moscou 33(1): 503 (1860).
≡ Philammos arbuscula (Pall.) Steven, Bull. Soc. Imp. Naturalistes Moscou 29(II): 146 (1856).
≡ Tragacantha arbuscula (Pall.) Kuntze, Revis. Gen. Pl. 2: 943 (1891).
= Astragalus arbuscula var. *leucotricha* Trautv., Bull. Soc. Imp. Naturalistes Moscou 33(1): 504 (1860).
= *Astagalus arbuscula* var. *microphyllus* Krylov & Serg., Fl. Zapadnoĭ Sibiri 7: 1695 (1933).
= *Astagalus eremothamnus* Kar. & Kir., Bull. Soc. Imp. Naturalistes Moscou 15: 334 (1842).
= *Astagalus horizontalis* Kar. & Kir., Bull. Soc. Imp. Naturalistes Moscou 14: 406 (1841).

###### Notes.

This species is distributed in China, Kazakhstan, Mongolia, and Russiain Asia (Podlech and Zarre 2013; Baasanmunkh et al. 2024b; POWO 2025). We found this species (voucher number: MT1-2) in semi-desert habitats of the Chu-Ily Mountain range, occurring at elevations of 500–550 m. The morphological characters of this species are very similar to *A. astrachanicus* Sytin & Laktionov, but it can be distinguished by its peduncles 7–10 cm long; racemes 2–3 cm, densely 8–22-lowered (vs. 3–3.5 cm long; racemes c. 14 cm, loosely 15–20-flowered), pods 20–30 mm, shortly acuminate, with ± appressed white and black hairs (vs. 18–20 mm, with a beak 3–5 mm, with ascending white hairs).

**Figure 19.**
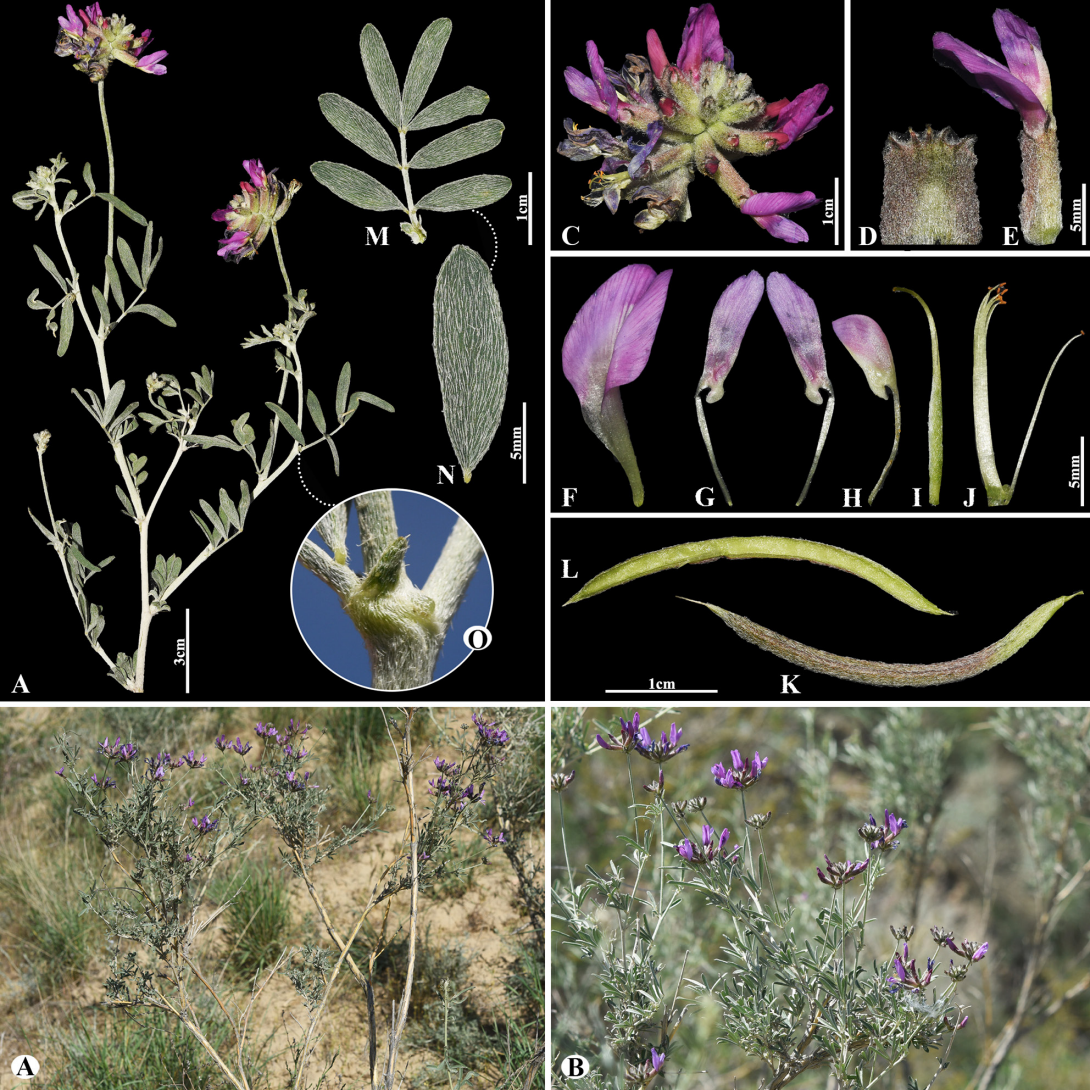
*Astragalus arbuscula* in Kazakhstan (Voucher number: MT1-2). **A**. General habit; **B**. Flowering branch; **C**. Raceme; **D**. Calyx; **E**. Flower; **F**. Standard; **G**. Wings; **H**. Keel; **I**. Pistil; **J**. Stamens; **K**. Pod; **L**. Pod valve; **M**. Leaf; **N**. Leaflet; **O**. Stipules. (Photo credits: D.Munkhtulga).

##### Astragalus macrotropis

Taxon classification

Plantae

FabalesFabaceae

Bunge, Mém. Acad. Imp. Sci. Saint Pétersbourg, Sér. 7, 11(16): 127 (1868).

CDD4650E-1A3A-5914-AC28-644E3DB2CB58

Fig. 20

≡ Philammos macrotropis (Bunge) Nevski, Trudy Bot. Inst. Akad. Nauk S.S.S.R., Ser. 1, Fl. Sist. Vyssh. Rast. 4: 252 (1937).
≡ Tragacantha macrotropis (Bunge) Kuntze, Revis. Gen. Pl. 2: 946 (1891).
= Astragalus stenoceras var. *macranthus* Bunge, Bull. Soc. Imp. Naturalistes Moscou 39(2): 25 (1866).

###### Notes.

This species is distributed throughout Kazakhstan, Uzbekistan, Tajikistan, Kyrgyzstan, and northwestern China (Xinjiang) (Podlech and Zarre 2013). We found this species (voucher number: MT6-1) in the rocky and rubbly slopes of the Chu-Ily Mountain range, occurring at elevations of 850–900 m. The morphological characters of this species are very similar to that of *A. bossuensis* Popov but distinguished by having stipules 2.5–3.5 mm long, white hairy, sometimes with black hairs (vs. 3–5 mm long, covered with white and black hairs), leaves 3–7 cm long; petiole 1–2.5 cm long (vs. 4–12 cm long; petiole 1.5–4 cm long), petals pink, often yellowish-tinged (vs. greenish-yellow) and pods 25–40 mm long (vs. 12–20 mm long).

**Figure 20.**
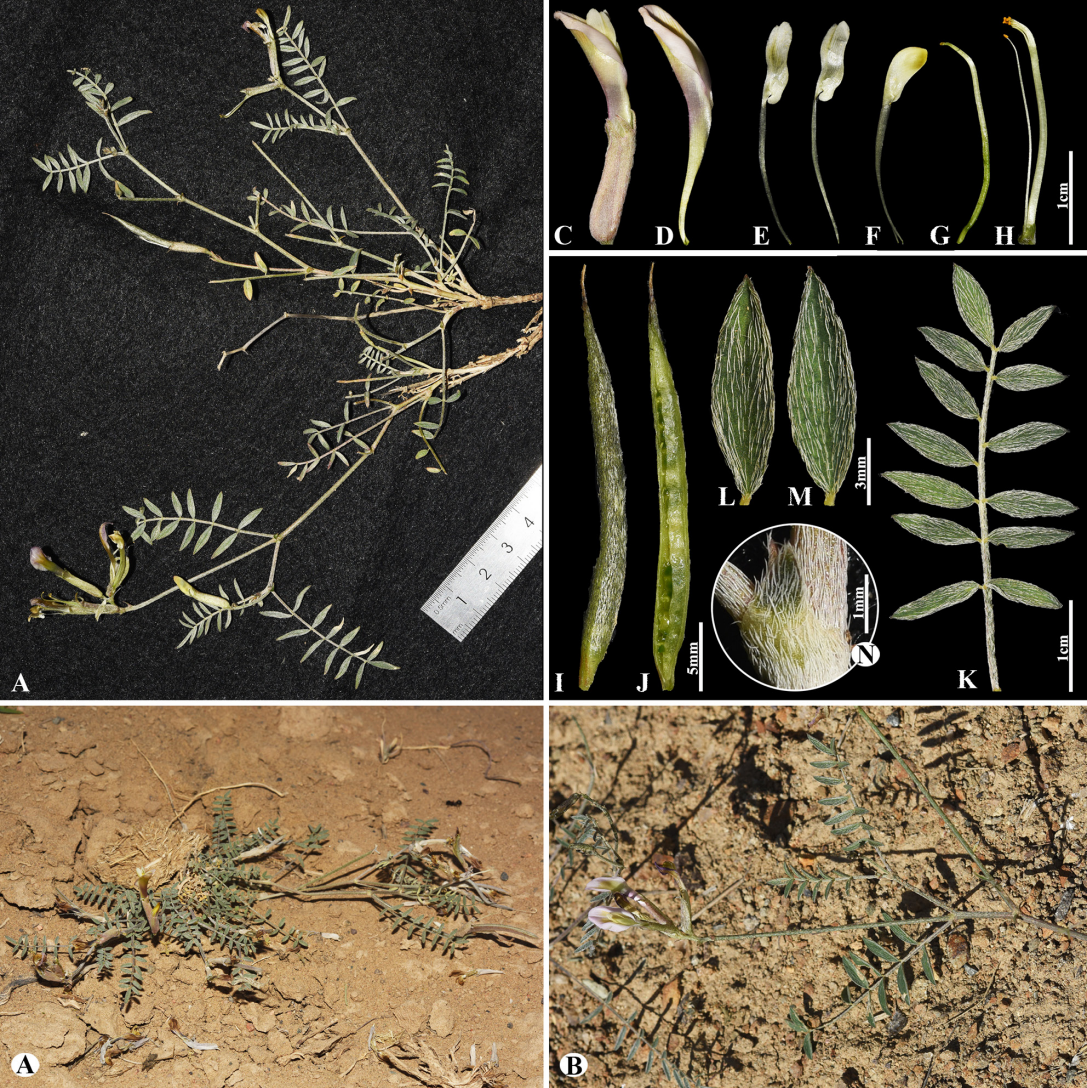
*Astragalus macrotropis* in Kazakhstan (Voucher number: MT6-1). **A**. General habit; **B**. Flowering branch; **C**. Flower; **D**. Standard; **E**. Wings; **F**. Keel; **G**. Pistil; **H**. Stamens; **I**. Pod; **J**. Pod valve; **K**. Leaf; **L**. Leaflet, adaxial view; **M**. Leaflet, abaxial view; **N**. Stipules. (Photo credits: D.Munkhtulga).

##### Astragalus platyphyllus

Taxon classification

Plantae

FabalesFabaceae

Kar. & Kir., Bull. Soc. Imp. Naturalistes Moscou 15: 345 (1842).

DCDA6706-61BF-5B5F-B337-ACE5851666E8

Fig. 21

≡ Chondrocarpus platyphyllus (Kar. & Kir.) Steven, Bull. Soc. Imp. Naturalistes Moscou 29(2): 149 (1856), not validly publ.
≡ Tragacantha platyphylla (Kar. & Kir.) Kuntze, Revis. Gen. Pl. 2: 947 (1891).
= Astragalus sykensis Freyn, Bull. Herb. Boissier, sér. 2, 5: 559 (1905).

###### Notes.

The native range of this species is Central Asia to Xinjiang (POWO 2025). We found this species (voucher number: MT3-3) in rocky and rubbly slopes of the Chu-Ily Mountain range, occurring at elevations of 850–900 m. The sectional placement of *A. platyphyllus* remains controversial. According to phylogenetic studies, including many species of the sect. *Incani*, this species has been excluded from *Incani* and placed in the sect. *Dissitiflori* (Amini et al. 2019). However, while the sect. *Dissitiflori* consists of shrubby, subshrubby, or occasionally herbaceous perennials with well-developed stems, *A. platyphyllus* is a perennial acaulescent species, making direct placement in the sect. *Dissitiflori* difficult. Therefore, more comprehensive molecular research, including representatives of the sect. *Dissitiflori*, are required to clarify its taxonomic position.

**Figure 21.**
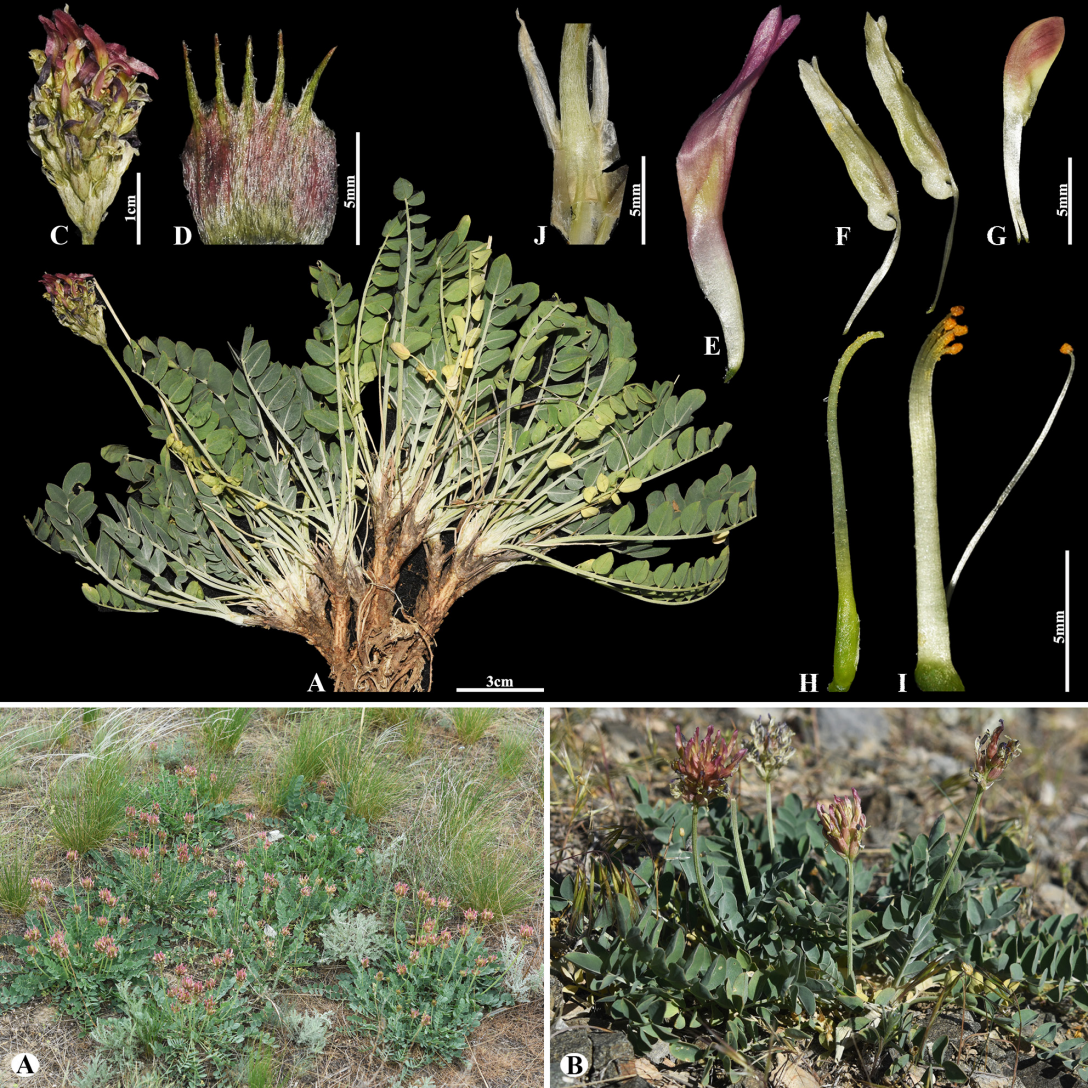
*Astragalus platyphyllus* in Kazakhstan (Voucher number: MT3-3). **A**. Habitat; **B**. General habit; **C**. Raceme; **D**. Calyx; **E**. Standard; **F**. Wings; **G**. Keel; **H**. Pistil; **I**. Stamens; **J**. Stipules. (Photo credits: D.Munkhtulga).

#### Sect. *Erioceras* Bunge, Mém. Acad. Imp. Sci. Saint Pétersbourg 11(16): 109 (1868).

##### Astragalus infractus

Taxon classification

Plantae

FabalesFabaceae

Sumnev., Sist. Zametki Mater. Gerb. Krylova Tomsk. Gosud. Univ. Kuybysheva 1936(9–10): 4 (1937).

093C9660-AD3A-5F01-B40A-9B03217D486D

Fig. 22

###### Notes.

This is a Central Asian endemic species restricted to the northern Tian Shan Mountains along the Kazakhstan–Kyrgyzstan border (Podlech and Zarre 2013). We found this species (voucher number: MT11-4) in rocky and stony areas of the Kurtogay, Kegen district, occurring at elevations of 1,400–1,450 m. *A. infractus* is morphologically similar to *A. ferganensis* (Popov) B.Fedtsch. ex Korol., but differs by its leaflet narrowly ovate to elliptic, 5–10 × 2.5–4 mm, shortly acuminate (vs. elliptic to obovate, 12–14 × 5–9 mm, minutely mucronulate) and stem 6–14 cm long (vs. 1–3(–6) cm long). It is also similar to *A. psilolobus* in leaf shape and general habit, but differs in its height 10–18 cm (vs. 5–8 cm, shortly caulescent), peduncles 2–4 cm long (vs. up to 1 cm) pods 30–40 mm, subsessile, incompletely bilocular, dense tomentose hairs (11–15 × 2–3 mm, sessile, ventrally keeled, glabrous or with scattered hairs).

**Figure 22.**
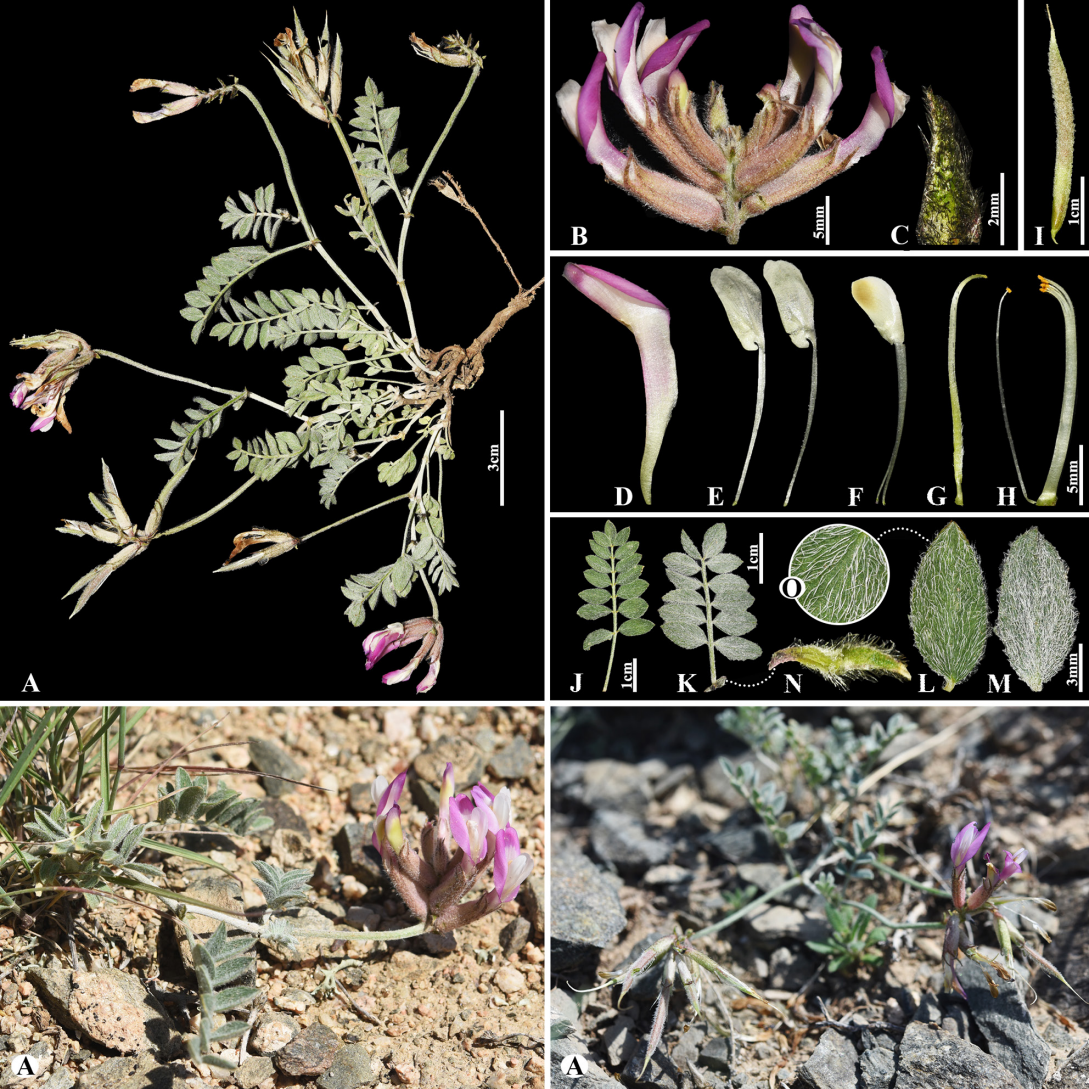
*Astragalus infractus* in Kazakhstan (Voucher number: MT11-4). **A**. General habit; **B**. Raceme; **C**. Bract; **D**. Standard; **E**. Wings; **F**. Keel; **G**. Pistil; **H**. Stamens; **I**. Pod; **J**. Leaf, adaxial view; **K**. Leaf, abaxial view; **L**. Leaf, adaxial view; **M**. Leaflet, abaxial view; **N**. Stipules; **O**. omission of medifixed hairs on the upper side of the leaf. (Photo credits: D.Munkhtulga).

##### Astragalus petraeus

Taxon classification

Plantae

FabalesFabaceae

Kar. & Kir., Bull. Soc. Imp. Naturalistes Moscou 15: 333 (1842).

77526D7D-7621-5778-94CD-B44AEEC970FF

Fig. 23

≡ Tragacantha petraea (Kar. & Kir.) Kuntze, Revis. Gen. Pl. 2: 947 (1891).
= Astragalus irkeschtamii B.Fedtsch., Bot. Mater. Gerb. Bot. Inst. Komarova Akad. Nauk S.S.S.R. 8: 168 (1940).
= Astragalus xylorrhizus Bunge, Izv. Imp. Obshch. Lyubit. Estestv. Moskovsk. Univ. 26(2): 267 (1880).

###### Notes.

This species is endemic to Central Asia and distributed throughout Kazakhstan, Kyrgyzstan, and Tajikistan (Podlech and Zarre 2013). We found this species (voucher number: MT11-6) in rocky, rock-rubbly, and arid slopes of the Kurtogay, Kegen district, occurring at elevations of 1,400–1,500 m. *A. petraeus* was morphologically similar to *A. arcuatus* Kar. & Kir., but differs by racemes with 10–18 flowers, orbicular-ovate (vs. with 5–7 flowers, capitate), leaflets 4–7 pairs, elliptical or oval (vs. 2–3(–4) pairs, lanceolate or linear-lanceolate).

**Figure 23.**
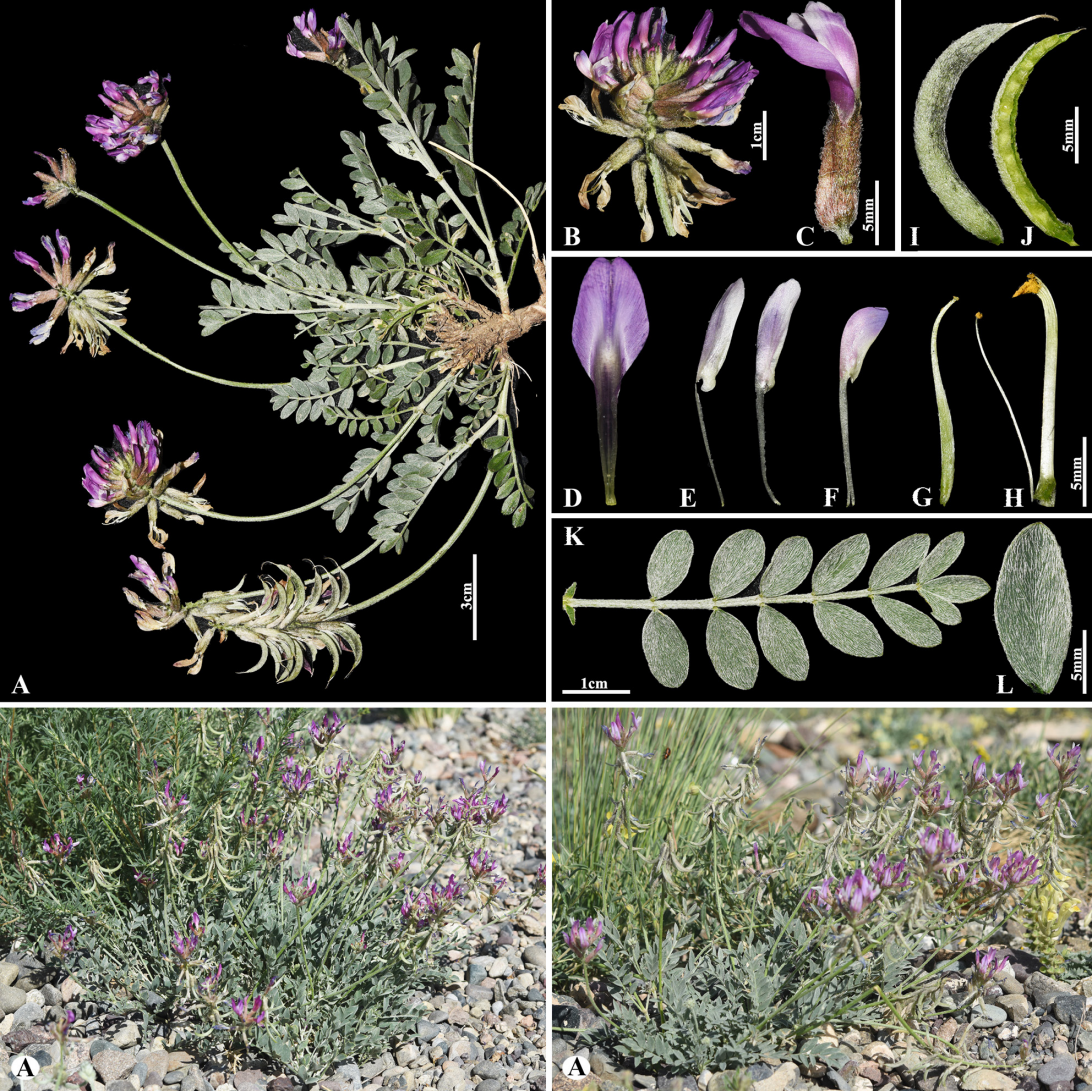
*Astragalus petraeus* in Kazakhstan (Voucher number: MT11-6). **A**. General habit; **B**. Raceme; **C**. Flower; **D**. Standard; **E**. Wings; **F**. Keel; **G**. Pistil; **H**. Stamens; **I**. Pod; **J**. Pod valve; **K**. Leaf; **L**. Leaflet. (Photo credits: D.Munkhtulga).

##### Astragalus turczaninowii

Taxon classification

Plantae

FabalesFabaceae

Kar. & Kir., Bull. Soc. Imp. Naturalistes Moscou 15: 342 (1842).

5FC83BDD-2ADE-5E5B-9328-3FAEE23C9EDC

Fig. 24

≡ Tragacantha turczaninowii (Kar. & Kir.) Kuntze, Revis. Gen. Pl. 2: 948 (1891).
= Astragalus turczaninowii f. *pubiflorus* Basil., Bot. Mater. Gerb. Glavn. Bot. Sada R.S.F.S.R. 4: 46 (1923).

###### Notes.

This species is distributed throughout Afghanistan, Kazakhstan, Turkmenistan, and Uzbekistan (Podlech and Zarre 2013). We found this species (voucher number: MT1-4) in fixed sands and sandhills of the Chu-Ily Mountain range, occurring at elevations of 500–550 m. *Astragalus turczaninowii* belonging to sect. *Erioceras*, this species is distinguished from other members of the section by its caulescent herbaceous perennial habit, the stems and leaves densely covered with simple and bifurcate spreading hairs, and its loose racemes bearing very remote 9–17 flowers.

**Figure 24.**
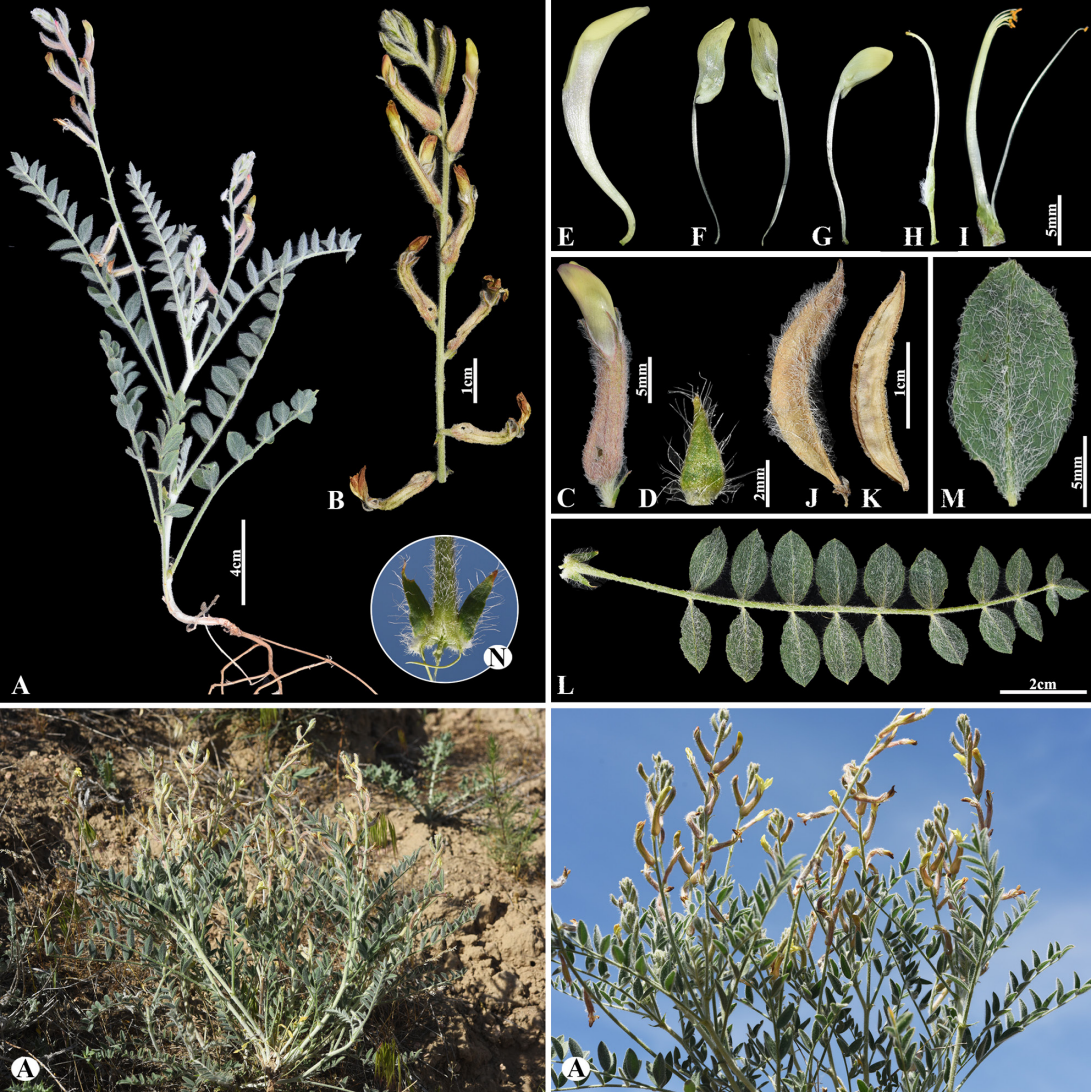
*Astragalus turczaninowii* in Kazakhstan (Voucher number: MT1-4). **A**. General habit; **B**. Raceme; **C**. Flower; **D**. Bract; **E**. Standard; **F**. Wings; **G**. Keel; **H**. Pistil; **I**. Stamens; **J**. Pod; **K**. Pod valve; **L**. Leaf; **M**. Leaflet; **N**. Stipules. (Photo credits: D.Munkhtulga).

#### Sect. *Laguropsis* Bunge, Mém. Acad. Imp. Sci. Saint Pétersbourg 11(16): 137 (1868).

##### Astragalus schrenkianus

Taxon classification

Plantae

FabalesFabaceae

Fisch. & C.A.Mey., Bull. Cl. Phys.-Math. Acad. Imp. Sci. Saint-Pétersbourg 2: 197 (1844).

E275B96F-7411-5D56-9FFF-22CAA7AC0442

Fig. 25

≡ Tragacantha schrenkiana (Fisch. & C.A.Mey.) Kuntze, Revis. Gen. Pl. 2: 948 (1891).
= Astragalus dschanbulakensis B.Fedtsch. in Trudy Imp. S.-Peterburgsk. Bot. Sada 24: 236 (1905).
= Astragalus holargyreus Bunge in Beitr. Fl. Russl.: 97 (1852).
= Astragalus nobilis var. dschanbulakensis (B.Fedtsch.) Basil. in Bot. Mater. Gerb. Glavn. Bot. Sada R.S.F.S.R. 3: 114 (1922).
= Astragalus turlanicus Bajtenov & Myrz. in Bot. Mater. Gerb. Bot. Inst. Bot. Acad. Nauk Kazakhsk. S.S.R. 10: 29 (1977).

###### Notes.

This species is endemic to Central Asia and occurs in Kazakhstan, Kyrgyzstan, and Uzbekistan (Podlech and Zarre 2013). We found this species (voucher number: MT2-2) in rocky and on rock-rubbly slopes of the Chu-Ily Mountain range, occurring at elevations of 800–950 m. *A. schrenkianus* is morphologically close to *A. schachimardanus* Basil., but differs by its leaflets in 3–5 pairs, narrowly elliptic to obovate, 8–20 × 3–7 mm (vs. in 4–7 pairs, very narrowly elliptic, 7–12 × 1.5–2 mm) and bracts only ciliate at the margins (vs. hairy on outer side).

**Figure 25.**
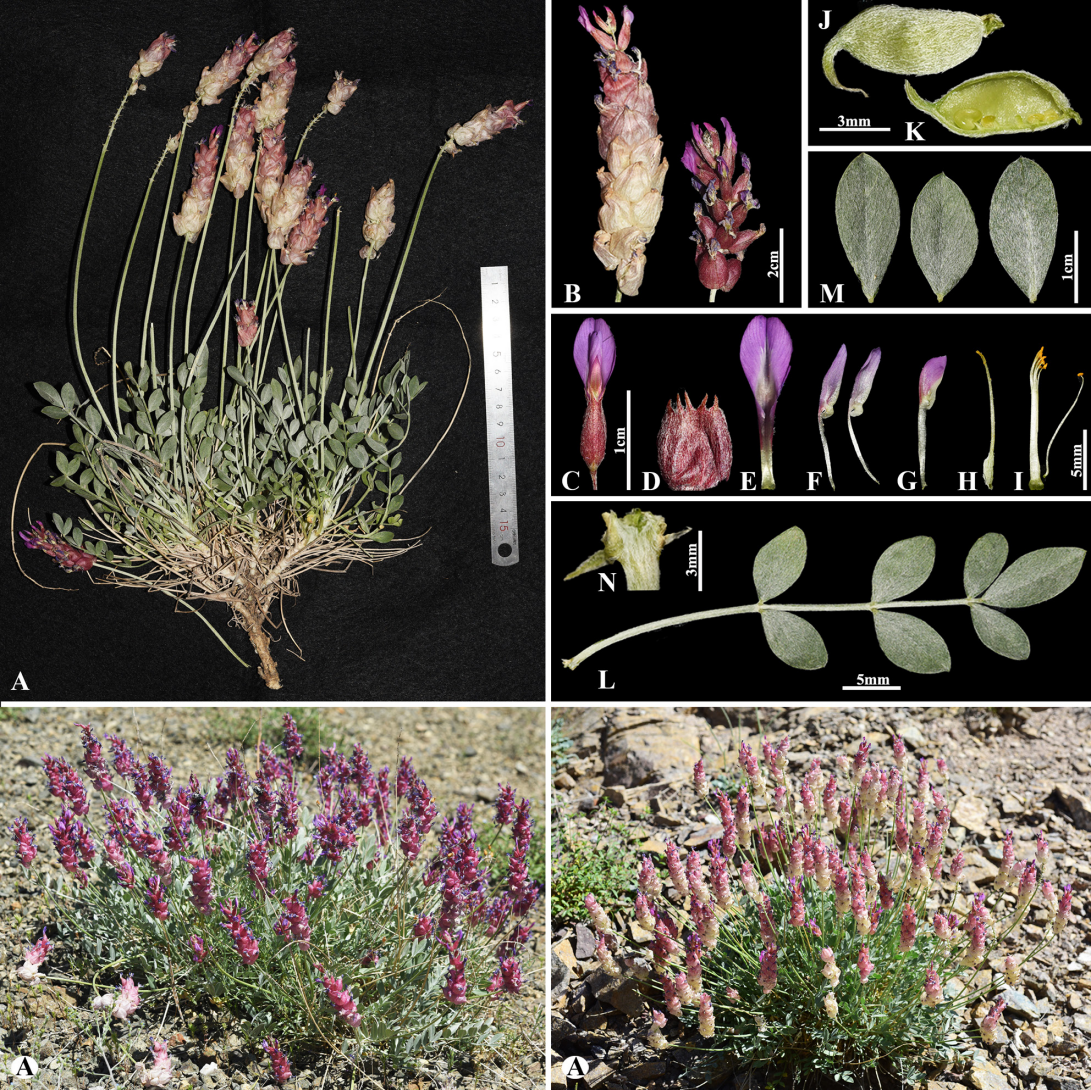
*Astragalus schrenkianus* in Kazakhstan (Voucher number: MT2-2). **A**. General habit; **B**. Raceme; **C**. Flower; **D**. Calyx; **E**. Standard; **F**. Wings; **G**. Keel; **H**. Pistil; **I**. Stamens; **J**. Pod; **K**. Pod valve; **L**. Leaf; **N**. Stipules. (Photo credits: D.Munkhtulga).

#### Sect. *Paracystium* Gontsch. in Fl. URSS 12: 881 (1946).

##### Astragalus lasiophyllus

Taxon classification

Plantae

FabalesFabaceae

Ledeb., Fl. Ross. 1: 627 (1843).

7D53484A-2160-58FC-9D90-25F2E2095EBC

Fig. 26

= *Astagalus inderiensis* Claus, Beitr. Pflanzenkunde Russ. Reiches Lief. 8: 64. (1851).

###### Notes.

This species is distributed in European-Russia, Kazakhstan, Uzbekistan, NW. China, and Mongolia (Podlech and Zarre 2013). We found this species (voucher number: MT10-2) in the rocky and rubbly slopes of the Balkhash Lake, Moiynkum district, occurring at elevations of 300–400 m. The name *A. pallasii* Biehler has been frequently misapplied (auct. non Biehler: see Podlech and Zarre 2013). According to Knyazev and Laktionov (2024), these specimens should be recognized as *A. lasiophyllus*, and it is appropriate to continue using this name.

**Figure 26.**
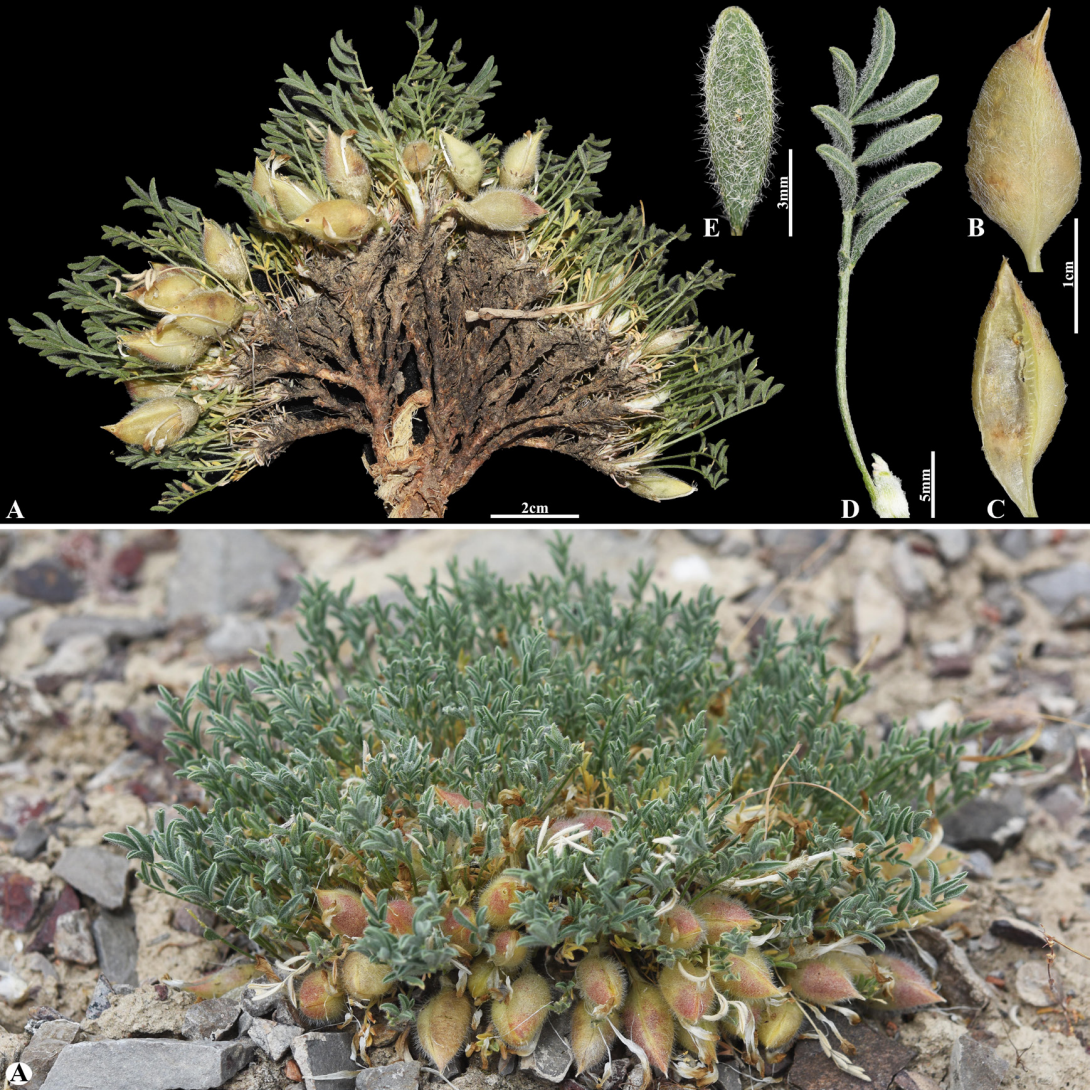
*Astragalus lasiophyllus* in Kazakhstan (Voucher number: MT10-2). **A**. General habit; **B**. Pod; **C**. Pod valve; **D**. Leaf; **E**. Leaflet. (Photo credits: D.Munkhtulga).

#### Sect. *Trachycercis* Bunge, Mém. Acad. Imp. Sci. Saint Pétersbourg 11(16): 114 (1868).

##### Astragalus borodinii

Taxon classification

Plantae

FabalesFabaceae

Krasn., Bot. Zap. 2(1): 15 (1887 publ. 1888).

91B8FE04-BD3A-5890-A564-FD2D32CA9110

Fig. 27

= Astragalus projecturus Sumnev., Sist. Zametki Mater. Gerb. Krylova Tomsk. Gosud. Univ. Kuybysheva 1936(9–10): 6 (1937).

###### Notes.

This species is distributed throughout Kazakhstan, Kyrgyzstan, Mongolia, and NW China (Podlech and Zarre 2013). We found this species (voucher number: MT11-2) in the rocky and rubbly slopes of the Kurtogay, Kegen district, occurring at elevations of 1,400–1,500 m. According to Ulziykhutag (2003), *A. borodinii* belongs to the sect. *Borodiniana*. Podlech and Zarre (2013) later transferred this section to the sect. *Trachycercis*. *A. borodinii* resembles *A. vallestris* Kamelin in terms of overall height; pinkish-white flowers; medifixed, appressed hairs on both leaflet surfaces. It differs from *A. vallestris* in that its leaves 2–5 cm long, (1–)2 pairs of leaflets, obovate, 8–20 × 3–8 mm, obtuse to subacute (vs. 1–2.5 cm long, only one leaflet, narrowly elliptic to elliptic, 6–18 × 2–6 mm long, acute).

**Figure 27.**
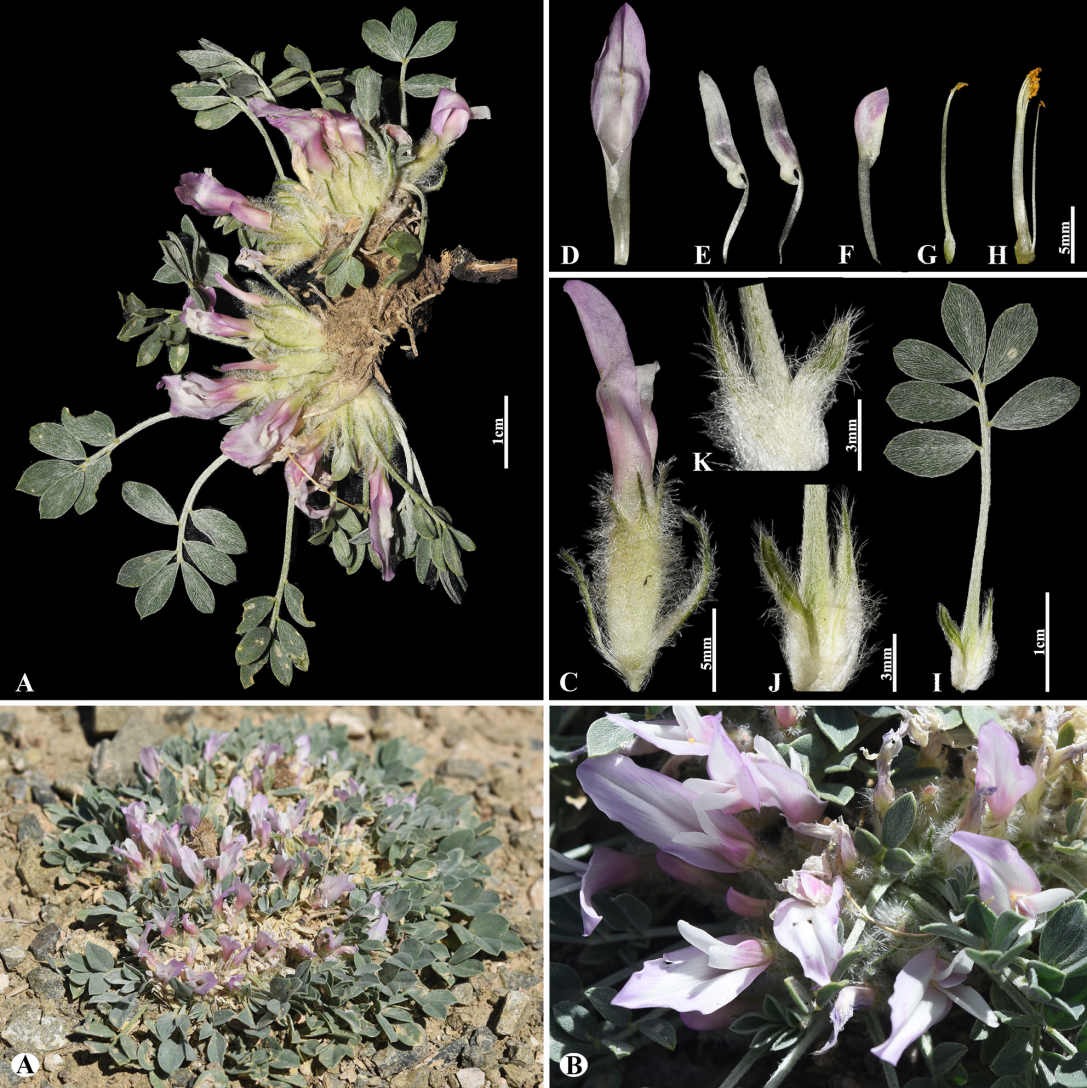
*Astragalus borodinii* in Kazakhstan (Voucher number: MT11-2). **A**. General habit; **B**. Flowers; **C**. Flower with bracts; **D**. Standard; **E**. Wings; **F**. Keel; **G**. Pistil; **H**. Stamens; **I**. Leaf; **J**. Stipules, inside view; **K**. Stipules, outside view. (Photo credits: D.Munkhtulga).

##### Astragalus scabrisetus

Taxon classification

Plantae

FabalesFabaceae

Bong., A.G.H.Bongard & C.A.Meyer, Verz. Saisang-Nor Pfl.: 89 (1841).

FA2F987B-3550-5F6B-BE55-3B27B9DB17AA

Fig. 28

≡ Ailuroschia scabriseta (Bong.) Steven, Bull. Soc. Imp. Naturalistes Moscou 29(2): 151 (1856).
≡ Tragacantha scabriseta (Bong.) Kuntze, Revis. Gen. Pl. 2: 948 (1891).

###### Notes.

This species is distributed throughout Kazakhstan, Uzbekistan, China (Xinjiang) and Mongolia (Podlech and Zarre 2013). We found this species (voucher number: MT0) in sandy, sandy-rocky, and rocky slopes of the Chu-Ily Mountain range, occurring at elevations of 900–1000 m. Morphological characteristics of this species very similar to *A. grubovii* Sanchir but distinguished by having leaflets 3–8 (–9) pairs, elliptical or obovate, subobtuse, more rarely subacute, 5–10 (–14) mm long, 2.5–8 mm broad, with dense, appressed, stiff hairs on both surfaces (vs. 6–13 pairs, suborbicular, orbicular-oval or orbicularovate, obtuse, 5–10 (–13) mm long, 3–7 (–10) mm broad, with densely appressed or semipatent stiff hairs on both surfaces).

**Figure 28.**
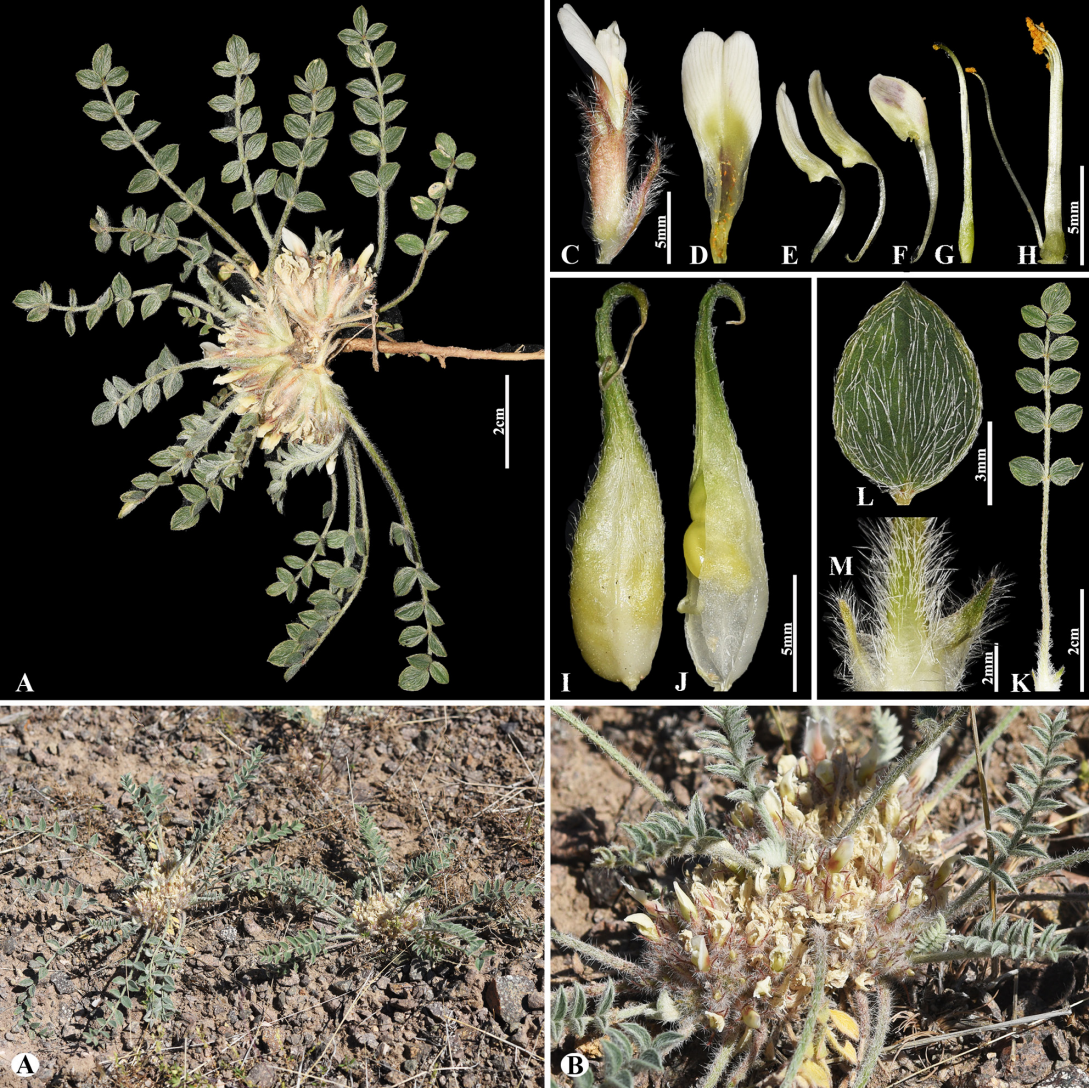
*Astragalus scabrisetus* in Kazakhstan (Voucher number: MT0). **A**. General habit; **B**. Flowers; **C**. Flower with bract; **D**. Standard; **E**. Wings; **F**. Keel; **G**. Pistil; **H**. Stamens; **I**. Pod; **J**. Pod valve; **K**. Leaf; **L**. Leaflet; **M**. Stipules. (Photo credits: D.Munkhtulga).

#### *Astragalus* subgenus *Trimeniaeus* Bunge, Astrag. geront. 1: 6 (1868).

**Sect. *Oxyglottis* Bunge, Mém. Acad. Imp. Sci. Saint Pétersbourg 11(16): 10 (1868)**.

##### Astragalus oxyglottis

Taxon classification

Plantae

FabalesFabaceae

Steven ex M.Bieb., Fl. Taur.-Caucas. 2: 192 (1808).

5FB64C0F-4D5E-5D04-AA6C-FE1DC500350C

Fig. 29

≡ Tragacantha oxyglottis (Steven ex M.Bieb.) Kuntze, Revis. Gen. Pl. 2: 947 (1891).
= *Astagalus psiloglottis * DC., Prodr. 2: 288 (1825).
= *Astagalus abbas-riazi* Parsa, Fl. Iran 9: 6 (1966).

###### Notes.

This species is distributed throughout Southern and Eastern Europe, Western Asia (including the Near East and the Caucasus), Central Asia, and South to East Asia (Podlech and Zarre 2013). We found this species (voucher number: MT5-9) in sandy soils of the Kanshengel, Zhambyl district, occurring at elevations of 400–420 m. Morphologically, this species is most similar to *A. vicarius* Lipsky but differs in several characteristics: stipules 2–4 mm long (vs. 3–6 mm long); calyx 2–2.5 mm long (vs. 3–4 mm long); pods narrowly ovoid, quadrangular in cross-section, sharply keeled, with thin tough valves, glabrous or hairy (vs. narrowly ovate-acuminate, triangular in cross-section, dorsally flattened, with winglike dentate crest along margins, valves thin, straw-colored, glabrous).

**Figure 29.**
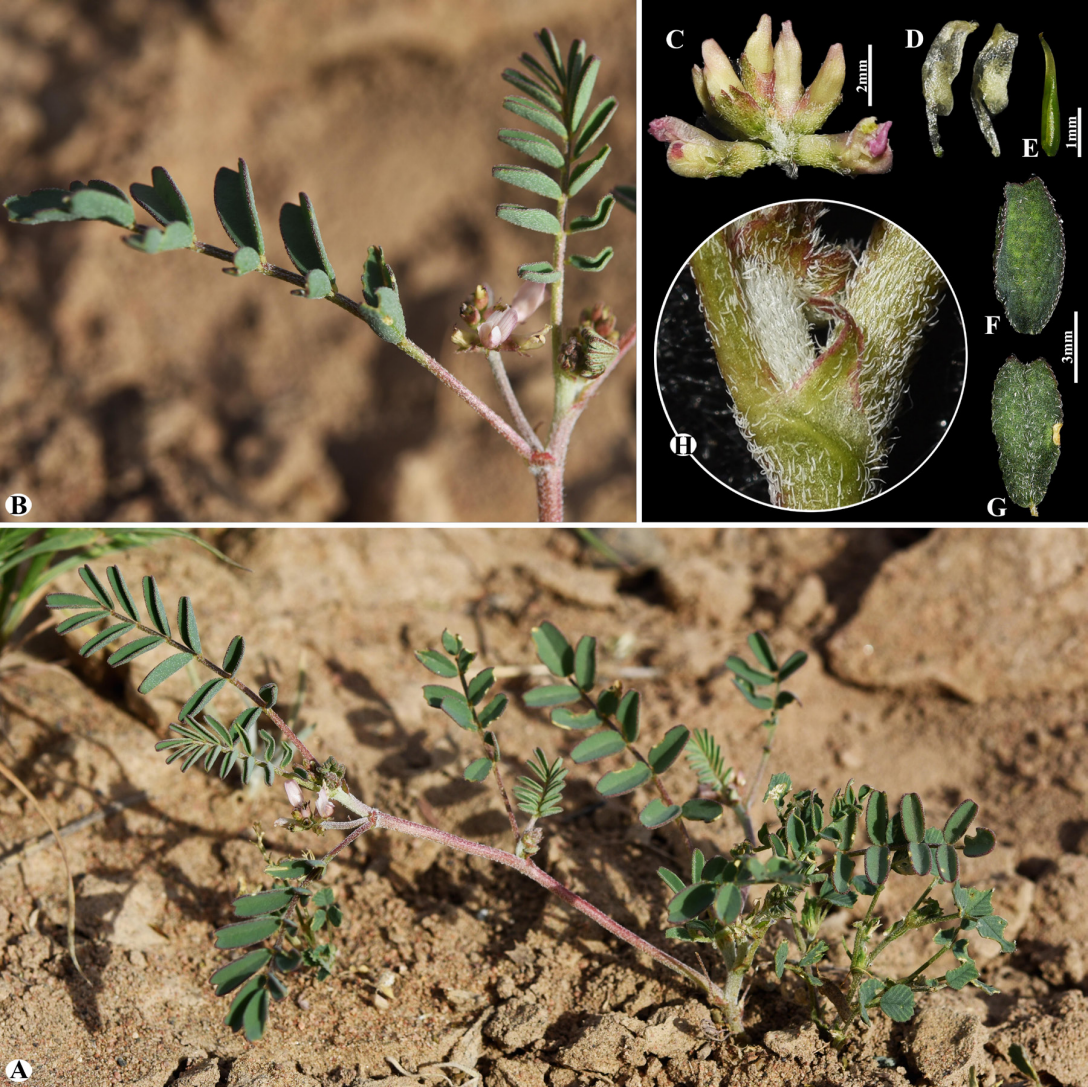
*Astragalus oxyglottis* in Kazakhstan (Voucher number: MT5-9) **A**. General habit; **B**. Flowering branch; **C**. Flower; **D**. Wings; **E**. Pistil; **F**. Leaf, adaxial view; **G**. Leaf, abaxial view; **H**. Stipules. (Photo credits: D.Munkhtulga).

## Conclusion

In this study, we investigated the taxonomic and phylogenetic relationships of 26 *Astragalus* species that comprise only 8% of the total *Astragalus* (approximately 320 species) in Kazakhstan (Abdulina 1998; Perezhogin et al. 2023). According to our phylogenetic results, all the studied species were divided into two clades, and some species showed relationships consistent with previous morphology-based classifications (Podlech and Zarre 2013), whereas others appeared in different phylogenetic positions. The phylogenetic tree provides a preliminary framework for understanding the relationships among *Astragalus* species across Central Asia and emphasizes the importance of broader datasets to achieve stable sectional delimitation. While these findings suggest that current taxonomic boundaries may require revision, we refrain from proposing formal reclassification due to limited sampling and genetic resolution. Further integrative research combining multiple nuclear and plastid markers with detailed morphological and biogeographic analyses is essential to refine the infrageneric taxonomy of *Astragalus*. The detailed illustrations and species documentation presented in this study will serve as significant references for future taxonomic and identification studies of *Astragalus* genus.

## Supplementary Material

XML Treatment for Astragalus balchaschensis

XML Treatment for Astragalus flexus

XML Treatment for Astragalus lasiopetalus

XML Treatment for Astragalus macronyx

XML Treatment for Astragalus lanuginosus

XML Treatment for Astragalus mucidus

XML Treatment for Astragalus vulpinus

XML Treatment for Astragalus sphaerophysa

XML Treatment for Astragalus sieversianus

XML Treatment for Astragalus brachypus

XML Treatment for Astragalus paucijugus

XML Treatment for Astragalus chaetodon

XML Treatment for Astragalus psilolobus

XML Treatment for Astragalus kronenburgii

XML Treatment for Astragalus pseudocytisoides

XML Treatment for Astragalus arbuscula

XML Treatment for Astragalus macrotropis

XML Treatment for Astragalus platyphyllus

XML Treatment for Astragalus infractus

XML Treatment for Astragalus petraeus

XML Treatment for Astragalus turczaninowii

XML Treatment for Astragalus schrenkianus

XML Treatment for Astragalus lasiophyllus

XML Treatment for Astragalus borodinii

XML Treatment for Astragalus scabrisetus

XML Treatment for Astragalus oxyglottis

## Additional information

### Artificial Intelligence (AI) use

The authors accept full responsibility for the content of the manuscript, including the disclosure of any use of AI.

No AI tools were used in the preparation of this manuscript.

### Author ORCIDs

D. Munkhtulga https://orcid.org/0009-0009-0175-203X

S. Baasanmunkh https://orcid.org/0000-0003-4224-9376

J.H. Park https://orcid.org/0009-0003-9053-8591

N. Nyamgerel https://orcid.org/0000-0002-9110-423X

S. Kubentayev https://orcid.org/0000-0002-0369-0591

D. Alibekov https://orcid.org/0000-0003-1555-1430

S. Mukhtubayeva https://orcid.org/0000-0001-5921-3113

Z. Idrissova https://orcid.org/0000-0003-3231-8518

A. Urazalina https://orcid.org/0000-0002-0319-6577

H.J. Choi https://orcid.org/0000-0001-6315-0071

### Data availability

All of the data that support the findings of this study are available in the main text or Supplementary Information.

## Supplementary materials

Supplementary material 1 Species list Data type xlsx Explanation note Detailed information on species, section, subgenus name, GenBank accession numbers, and references of the samples used in this study. https://binary.pensoft.net/file/1628908This dataset is made available under the Open Database License (http://opendatacommons.org/licenses/odbl/1.0/). The Open Database License (ODbL) is a license agreement intended to allow users to freely share, modify, and use this Dataset while maintaining this same freedom for others, provided that the original source and author(s) are credited. Dariganga Munkhtulga, Shukherdorj Baasanmunkh, Jong Ho Park, Nudkhuu Nyamgerel, Serik Kubentayev, Daniyar Alibekov, Saule Mukhtubayeva, Zhansaya Idrissova, Alina Urazalina, Hyeok Jae Choi
